# Glycated Hemoglobin as a Marker for Predicting Outcomes of Patients With Stroke (Ischemic and Hemorrhagic): A Systematic Review and Meta-Analysis

**DOI:** 10.3389/fneur.2021.642899

**Published:** 2021-03-31

**Authors:** Yaya Bao, Dadong Gu

**Affiliations:** ^1^Shaoxing University Medical College, Shaoxing, China; ^2^Department of Neurology, Zhuji Affiliated Hospital of Shaoxing University, Zhuji, China

**Keywords:** glycated hemoglobin, stroke, glycemic control, ischemic stroke, meta-analysis

## Abstract

**Background:** Glycated hemoglobin (HbA1c) has emerged as a useful biochemical marker reflecting the average glycemic control over the last 3 months, and the values are not affected by short-term transient changes in blood glucose levels. However, its prognostic value in the acute neurological conditions such as stroke is still not well-established. The present meta-analysis was conducted to assess the relationship of HbA1c with outcomes such as mortality, early neurological complications, and functional dependence in stroke patients.

**Methods:** A systematic search was conducted for the PubMed, Scopus, and Google Scholar databases. Studies, either retrospective or prospective in design that examined the relationship between HbA1c with outcomes of interest and presented the strength of association in the form of adjusted odds ratio/hazard ratios were included in the review. Statistical analysis was done using STATA version 13.0.

**Results:** A total of 22 studies (15 studies on acute ischemic stroke and seven studies on hemorrhagic stroke) were included in the meta-analysis. For patients with acute ischemic stroke, each unit increase in HbA1c was found to be associated with an increased risk of mortality within 1 year, increased risk of poor functional outcome at 3 months, and an increased risk of symptomatic intracranial hemorrhage (sICH) within 24 h of admission. In those with HbA1c ≥ 6.5%, there was an increased risk of mortality within 1 year of admission, increased risk of poor functional outcomes at 3 and 12 months as well as an increased risk of symptomatic intracranial hemorrhage (sICH) within 24 h of admission. In patients with hemorrhagic stroke, each unit increase in HbA1c was found to be associated with increased risk of poor functional outcome within the first 3 months from the time of admission for stroke. In those with HbA1c ≥ 6.5%, there was an increased risk of poor functional outcome at 12 months.

**Conclusions:** The findings indicate that glycated hemoglobin (HbA1c) could serve as a useful marker to predict the outcomes in patients with stroke and aid in the implementation of adequate preventive management strategies at the earliest.

## Introduction

Diabetes mellitus is an increasingly growing medical condition that is estimated to affect nearly 400 million people globally as per the year 2015 estimates (1). Studies have predicted that by the year 2040, around 600 million people would have this chronic disease (1, 2). It is suggested that a substantial proportion of patients with stroke may have comorbid diabetes mellitus, and this is because diabetes is a well-established risk factor for neurovascular disease (3, 4). A recent meta-analysis found a significant association of acute hyperglycemia and diabetes with poor outcomes after stroke, both ischemic and hemorrhagic (5). Using around 27,000 subjects, a large multi-centric study found diabetes to be present in one-fifth of patients with acute stroke, whereas this proportion was less (22%) in those with no stroke (6). Further, the study also noted a higher magnitude of association of diabetes with ischemic stroke, as against hemorrhagic stroke (6).

Studies have shown that the presence of diabetes is associated with increased death, duration of stay at hospital, rates of readmission and poorer post-stroke functional and recovery outcomes (7–10). On the other hand, there are studies that have observed no substantial variations between subjects with or without diabetes in post-stroke outcomes (11, 12). Glycated hemoglobin (HbA1c) has emerged as a useful biochemical marker reflecting the average glycemic control over the last 3 months or 120 days (13, 14). The added advantage is that the possibility of misdiagnosis due to stress hyperglycemia is greatly reduced, and also, the values are not affected by short-term transient changes in blood glucose levels (13, 14). The measurement of HbA1c does not require overnight fasting, and the amount of blood required is also small (14). These characteristics probably make the testing for HbA1c feasible for routine screening of diabetes mellitus, particularly in hospital-based settings.

HbA1c has been shown to be a biochemical marker and a good predictor of vascular disruption is patients with diabetes (15, 16). It has also been shown to associate well with diabetic complications (15–17). However, its prognostic value in the acute neurological conditions such as stroke is still not well-substantiated. Studies have attempted to document the relationship of HbA1c levels and outcomes of patients with both ischemic and hemorrhagic stroke. However, the current understanding is not enough to inform the guidelines. There is a need for high-quality evidence through pooling of findings of studies in order to make an informed decision on the use of HbA1c for prediction of outcomes of stroke patients. With these considerations, the current meta-analysis was conducted to assess the relationship of HbA1c and outcomes (mortality, early neurological and functional) of stroke patients, both ischemic and hemorrhagic.

## Materials and Methods

### Search Strategy

The study was designed and conducted based on PRISMA (Preferred Reporting Items for Systematic Reviews and Meta-analyses) guidelines. A systematic search of English language papers published until November 30, 2020 was carried out through PubMed, Scopus, and Google academic databases. The search strategy included medical topic heading (MeSH) terminology and free text words. Supplementary Table 1 includes the details of the search strategy used. The literature search was directed toward identifying studies that reported on the association of HbA1c levels with mortality and/or functional outcomes and/or neurological complications in patients with stroke, either acute ischemic or hemorrhagic.

### Selection Criteria and Methods

Two subject experts from the team reviewed the studies identified on literature search. The titles and abstracts were screened as a first step, after elimination of the duplicates. The full text of possible studies was subsequently reviewed. Any disagreements in the inclusion of the studies were resolved through discussions between the study authors. Only the studies that complied with the inclusion criteria were chosen for the meta-analysis. For additional studies, the bibliographic list of included studies and related reviews on the subject were reviewed.

#### Inclusion Criteria

Studies that were either retrospective record-based study or prospective in design were considered for inclusion. For a study to be included in the meta-analysis, it should have examined the relationship between HbA1c with outcomes of interest (i.e., mortality, functional dependence, symptomatic intracranial hemorrhage, and neurological complications). Further, the study should also have reported on the strength of association in the form of adjusted odds ratio/hazard ratios.

#### Exclusion Criteria

Studies such as case-reports or review articles were excluded. Also, those studies that did not provide data on the outcomes of interest or did not present an adjusted estimate of association between HbA1c and the outcomes were excluded.

### Data Extraction and Quality Assessment

Two authors separately extracted relevant data from the included studies using a data extraction sheet. Data extracted from qualifying studies mainly included the study identifier, i.e., the name of the first author along with the year the research was conducted; study setting, i.e., the country where the study was carried out; and other aspects of the study such as the design, subject characteristics, overall sample size, exposure variable of interest, and the main findings. The quality assessment of the included studies was done through the use of Newcastle–Ottawa Quality Assessment Scale, which has been adapted for use in observational studies (18).

### Statistical Analysis

This meta-analysis, using STATA version 16.0, reported effect sizes as pooled odds ratio with 95% CI (confidence intervals). Analysis was done for acute ischemic stroke and hemorrhagic stroke separately. Subgroup analysis was done based on different reported cutoff of HbA1c. *I*^2^ was used as a measure to denote heterogeneity, and in instances where the value of *I*^2^ exceeded 40%, random effects model was used. For reporting statistical significance, a *p*-value of <0.05 was considered. Egger's test was employed to assess for presence or absence of publication bias, and this was further supported by visual inspection of funnel plots.

## Results

### Selection of Articles, Study Characteristics, and Quality of Included Studies

**Figure 1:**
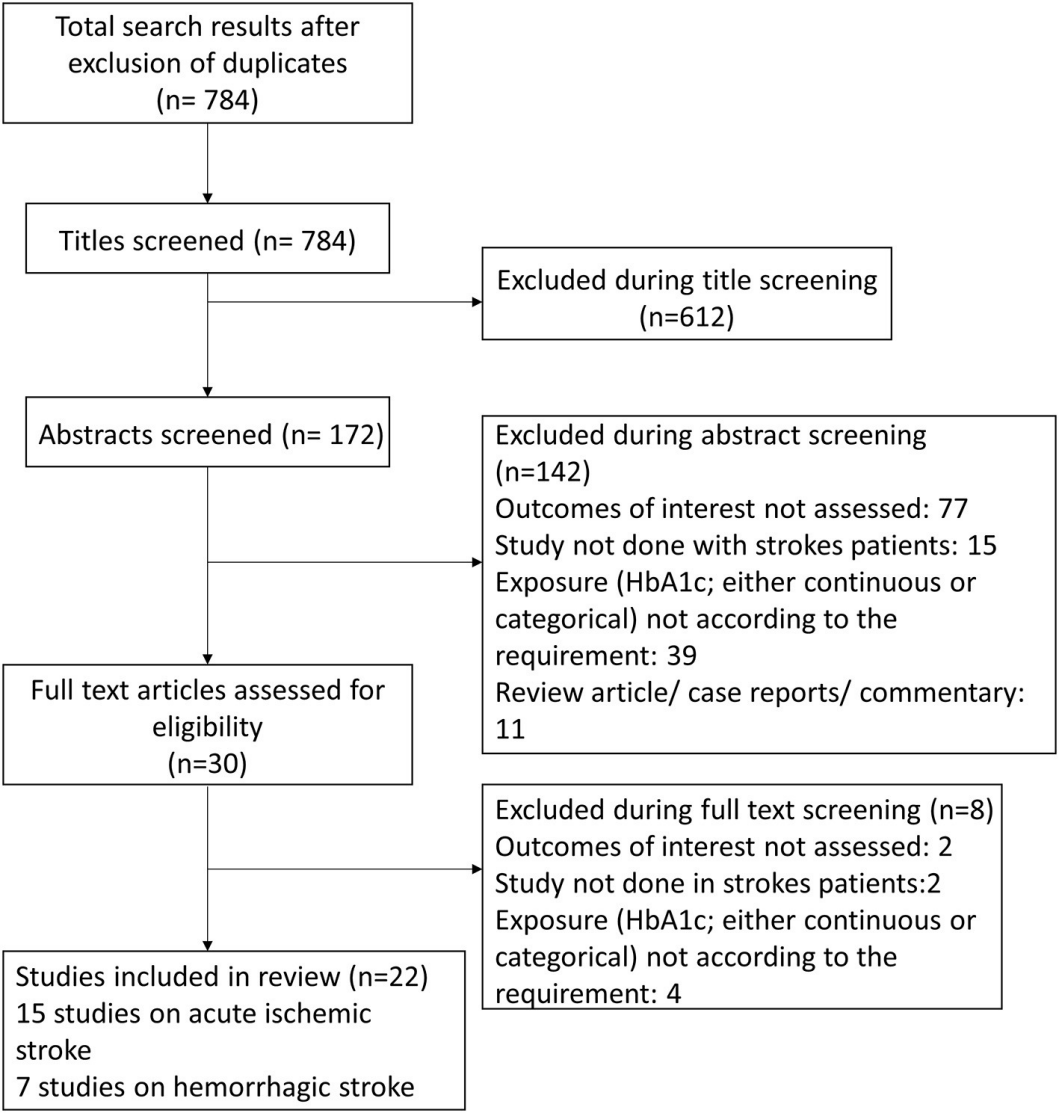
Selection process of the studies included in the review.

Using the search strategy and after removal of the duplicates, overall, 784 citations were obtained (Figure 1). Screening of the titles led to the removal of 612 studies. Out of the remaining 172 citations, 142 were omitted after reading the abstract. The remaining 30 papers were reviewed in detail, and finally, 22 articles were included in the meta-analysis with 15 studies on acute ischemic stroke and seven studies on hemorrhagic stroke (19–40). Tables 1, 2 present the details of the included studies. Among the studies that included patients with acute ischemic stroke, majority were conducted in China (6/15). One study each was conducted in New Zealand, Taiwan, Italy, India, South Korea, Sweden, Japan, Germany, and USA. Among these studies, nearly half (8/15) had a prospective design, while the remaining had a retrospective design. For studies with acute hemorrhagic stroke patients, majority were done in China (5/7) and one study each in USA and Japan. All the studies on acute hemorrhagic stroke were prospective in design. Almost all the studies were done in elderly subjects aged above 60 years.

**Table 1:** Characteristics of the studies included in the meta-analysis (for acute ischemic stroke).

**References**	**Study design**	**Country**	**Participant characteristics**	**Sample size**	**Exposure variable of interest**	**Key outcome**
Diprose et al. (19)	Prospective follow-up	New Zealand	Patients undergoing endovascular thrombectomy for ischemic stroke Mean (SD) age: 64.5 (14.6) years; 61% males	223	HbA1c as continuous variable	3-month mortality: OR 1.26 (95% CI: 1.01, 1.57) 3-month functional dependence: OR 1.32 (95% CI: 1.04, 1.67) 24-h neurological deterioration: OR 1.16 (95% CI:0.95, 1.41) Successful reperfusion: OR 1.11 (95% CI: 0.83, 1.49) Symptomatic intracerebral hemorrhage: OR 1.33 (95% CI: 1.03, 1.71)
Jing et al. (20)	Prospective cohort study	China	Ischemic stroke patients enrolled in the “ACROSS-China” cohort Mean (SD) age of participants with no diabetes: 60.6 (12.8) years; 67% male participants Mean (SD) age of participants with HbA1c ≥ 6.5%: 64.2 (11.6) years; 68% male participants	853 (712 with no diabetes; 141 with diabetes diagnosed as HbA1c ≥ 6.5%)	HbA1c ≥ 6.5%	12-month mortality *(N = 853)*: OR 1.02 (95% CI: 0.85, 1.22) 12-month stroke recurrence *(N = 838)*: OR 1.03 (95% CI: 0.86, 1.24) 12-month poor functional outcome *(N = 837)*: OR 1.19 (95% CI:0.74, 1.91)
Wang et al. (21)	Prospective follow-up study	China	Patients with first-ever ischemic stroke were enrolled and followed-up for neurological outcome assessment at 3 months post-stroke. Total sample of 408 subjects with mean (SD) age of 63.8 (11.5) years and 64% males	Overall 408; Low HbA1c (153) Moderate HbA1c (126) High HbA1c (129)	Low (<5.7%); moderate (5.7–6.4%) and high (≥6.5%) HbA1c values HbA1c as continuous variable	Moderate HbA1c: 3-month poor functional outcome (*N* = 279): OR 1.79 (95% CI:1.01, 3.21) High HbA1c 3-month poor functional outcome (*N* = 282): OR 2.39 (95% CI:1.20, 4.74) Continuous HbA1c: 3-month poor functional outcome (*N* = 408): OR 1.38 (95% CI:1.08, 1.78)
Zhang et al. (22)	Prospective cohort	China	Patients with acute anterior circulation stroke; mean age was around 65 years and around 60% subjects were males	426 subjects	HbA1c as continuous variable	Symptomatic intracerebral hemorrhage: OR 1.29 (95% CI: 1.09, 1.53) Poor functional outcome; short term: OR 1.48 (95% CI:1.23, 1.79)
Yang et al. (23)	Retrospective observational study	Taiwan	Patients with acute ischemic stroke Mean age was around 64 years and around 62% subjects were males	309 subjects	HbA1c ≥ 7.0%	Mortality: OR 0.42 (95% CI: 0.16, 1.13) Poor functional outcome: OR 0.83 (95% CI: 0.51, 1.35) Neurological complications: OR 0.60 (95% CI: 0.35, 1.03)
Wu et al. (24)	Prospective cohort	China	Patients with acute ischemic stroke; mean age of subjects around 65 years and around 60% males	Total of 2,186 subjects	HbA1C categorized as: <5.5%; 5.5– <6.1%; 6.1– <7.2%, and ≥7.2%	Mortality within 1 year HbA1c 5.5–6.1% (*N* = 507*):* OR 1.07 (95% CI: 0.57, 2.01) HbA1c 6.1– <7.2% (*N* = 579*):* OR 1.01 (95% CI: 0.53, 1.86) HbA1c ≥ 7.2% (*N* = 560): OR 2.45 (95% CI: 1.30, 4.62)
						Poor functional outcome at 3 months HbA1c 5.5–6.1% (*N* = 507*):* OR 1.57 (95% CI: 1.06, 2.33) HbA1c 6.1– <7.2% (*N* = 579*):* OR 1.30 (95% CI: 0.87, 1.93) HbA1c ≥ 7.2% (*N* = 560): OR 1.36 (95% CI: 0.84, 2.19) Poor functional outcome at 12 months HbA1c 5.5–6.1% (*N* = 507): OR 1.05 (95% CI: 0.63, 1.75) HbA1c 6.1– <7.2% (*N* = 579): OR 0.88 (95% CI: 0.54, 1.46) HbA1c ≥ 7.2% (*N* = 560): OR 1.20 (95% CI: 0.66, 2.19)
Lattanzi et al. (25)	Retrospective cohort	Italy	Patients with acute ischemic stroke; mean age of subjects around 70 years and around 60% males	112 subjects	HbA1C categorized as: <7.0% and ≥7.0%	Poor functional outcome at 3 months HbA1c ≥7.0%: OR 6.22 (95% CI: 1.94, 19.98)
Sunanda et al. (26)	Prospective case control study	India	Patients with acute ischemic stroke; mean (SD) age of subjects 56.7 (12.9) years; 72% males	130 subjects	HbA1C categorized as: <7.0% and ≥7.0%	Poor functional outcome at 3 months HbA1c ≥ 7.0%: OR 19.4 (95% CI: 5.9, 63.2)
Choi et al. (27)	Prospective cohort	South Korea	Patients with acute ischemic stroke (large vessel occlusion) treated with mechanical thrombectomy; subjects around 69 years of age and nearly 50% male	534 subjects	HbA1C categorized as: <6.5% and ≥6.5%	Poor functional outcome at 3 months HbA1c ≥ 6.5%: OR 2.22 (95% CI: 1.43, 3.45) Mortality within 3 months HbA1c ≥ 6.5%: OR 4.32 (95% CI: 2.41, 7.75) Symptomatic intracerebral hemorrhage HbA1c ≥ 6.5%: OR 1.50 (95% CI: 0.68, 3.30) Early neurological deterioration HbA1c ≥ 6.5%: OR 2.11 (95% CI: 1.31, 3.38)
Hjalmarsson et al. (28)	Retrospective analysis of patient data	Sweden	Patients with acute ischemic stroke; subjects around 75 years of age	501 subjects	HbA1C categorized as: ≤6.0% and >6.0% Continuous HbA1C	Mortality within 12 months HbA1c > 6.0%: OR 3.40 (95% CI: 1.40, 8.22) Continuous HbA1c: OR 1.29 (95% CI: 1.03, 1.62) Poor functional outcome at 12 months HbA1c > 6.0%: OR 2.68 (95% CI: 1.14, 6.03)
Kamouchi et al. (29)	Both prospective and retrospective cohort; data from multicenter hospital-based registry	Japan	Patients with acute ischemic stroke; mean (SD) age of participants 69 (12) years and 37.7% were women	3,627 subjects	HbA1C categorized as: Excellent (<6.2%) Good (6.2% to <6.9%) Fair (6.9% to <8.4%) Poor (≥8.4%)	Short term outcomes Neurological deterioration: HbA1c ≥ 6.9%: OR 1.65 (95% CI: 1.31, 2.06) Mortality: HbA1c ≥ 6.9%: OR 1.20 (95% CI: 0.79, 1.84) Poor functional outcome: HbA1c ≥ 6.9%: OR 1.35 (95% CI: 1.16, 1.58) Additional findings: Neurological deterioration: HbA1c Good: OR 1.02 (95% CI: 0.70, 1.46) HbA1c Fair: OR 1.66 (95% CI: 1.12, 2.43) HbA1c Poor: OR 2.32 (95% CI: 1.39, 3.83) Mortality: HbA1c Good: OR 1.20 (95% CI: 0.60, 2.64) HbA1c Fair: OR 1.02 (95% CI: 0.46, 2.45) HbA1c Poor: OR 0.46 (95% CI: 0.16, 1.38) Poor functional outcome: HbA1c Good: OR 1.16 (95% CI: 0.90, 1.51) HbA1c Fair: OR 1.26 (95% CI: 0.94, 1.71) HbA1c Poor: OR 2.30 (95% CI: 1.56, 3.40)
Rocco et al. (30)	Retrospective single-center study	Germany	Patients with acute ischemic stroke Mean age of participants around 68 years and around 60% subjects were males	112 subjects	Continuous HbA1c	Symptomatic intracerebral hemorrhage (within 24 h): OR 10.3 (95% CI: 3.89, 27.3) 3 month-mortality: OR 1.45 (95% CI: 1.25, 1.69) 3 month-poor functional outcome: OR 1.31 (95% CI: 1.15, 1.46)
Masrur et al. (31)	Retrospective analysis using GWTG-stroke database	USA	Patients with acute ischemic stroke; Median age of 72 years and 50% females	72,909 subjects	HbA1c categorized as ≤ 6.5% and >6.5%	Symptomatic intracerebral hemorrhage (within 24h): OR 1.25 (95% CI: 1.07, 1.46) In hospital-mortality: OR 1.36 (95% CI: 1.21, 1.53) Poor functional outcome at discharge: OR 1.29 (95% CI: 1.19, 1.39)
Gao et al. (32)	Retrospective review of data from hospital-based registry	China	Patients with acute ischemic stroke; mean age of 62 years and 70% males	793 subjects	HbA1c categorized as <5.9%; 5.9–6.7% and ≥6.7%	3-month poor functional outcome HbA1c 5.9–6.7%: OR 1.63 (95% CI: 0.89, 2.98) HbA1c ≥ 6.7%: OR 2.10 (95% CI: 1.16, 3.79)
Lei et al. (33)	Chengdu stroke registry with prospective follow-up	China	Patients with acute ischemic stroke; mean age of 65 years and 60% males	1,877 subjects	HbA1c categorized as 4.7–6.7%; 6.8% to 8.2% and >8.2%	3-month poor functional outcome HbA1c 6.8–8.2%: OR 1.22 (95% CI: 0.86, 1.55) HbA1c >8.2%: OR 1.43 (95% CI: 1.15, 2.39) 3-month mortality HbA1c 6.8–8.2%: OR 1.32 (95% CI: 0.63, 3.01) HbA1c >8.2%: OR 1.43 (95% CI: 1.01, 1.98) 12-month poor functional outcome HbA1c 6.8–8.2%: OR 1.02 (95% CI: 0.52, 1.69) HbA1c >8.2%: OR 1.17 (95% CI: 1.01, 1.83) 12-month mortality HbA1c 6.8–8.2%: OR 1.22 (95% CI: 0.59, 1.65) HbA1c >8.2%: OR 1.48 (95% CI: 1.03, 2.30)

**Table 2:** Characteristics of the studies included in the meta-analysis (for acute hemorrhagic stroke).

**References**	**Study design**	**Country**	**Participant characteristics**	**Sample size**	**Exposure variable of interest**	**Key outcome**
Kang et al. (34)	Multicenter prospective observational cohort study	China	Patients with spontaneous intracranial hemorrhage (sICH)	1,515 subjects	HbA1c with following cut-offs <6.0%; 6.0–7.9%; ≥8.0%	3-month poor functional outcome HbA1c ≥ 6.0%: OR 0.94 (95% CI: 0.67, 1.32) HbA1c 6.0–7.9%: OR 0.79 (95% CI: 0.32, 1.97) HbA1c ≥ 8.0%: OR 0.69 (95% CI: 0.14, 3.30)
Dandapat et al. (35)	GWTG-Stroke prospective registry of patients with ICH	USA	Patients with spontaneous intracranial hemorrhage (sICH); mean age around 67 years and 45% females	75,455 subjects	HbA1c with following cut-offs <5.7%; 5.7–6.5%; 6.5–8.0%; >8.0%	In-hospital mortality HbA1c ≥ 6.5%: OR 0.91 (95% CI: 0.87, 0.95) HbA1c > 8.0%: OR 0.78 (95% CI: 0.65, 0.93) HbA1c 6.5–8.0%: OR 0.73 (95% CI: 0.62, 0.87) HbA1c 5.7–6.5%: OR 0.72 (95% CI: 0.60, 0.87) Poor functional outcome at discharge HbA1c ≥ 6.5%: OR 0.96 (95% CI: 0.93, 0.99) HbA1c > 8.0%: OR 0.78 (95% CI: 0.57, 1.06) HbA1c 6.5–8.0%: OR 0.75 (95% CI: 0.55, 1.02) HbA1c 5.7–6.5%: OR 0.79 (95% CI: 0.56, 1.10)
Koga et al. (36)	Prospective multicenter observational study	Japan	Patients with hyperacute ICH; mean age of around 65 years with 60% males	176 subjects	Continuous HbA1c (per unit increase)	3-month mortality OR 1.04 (95% CI: 0.46, 1.97) 3-month functional outcome OR 1.54 (95% CI: 0.90, 2.94)
Zhang et al. (37)	Nationwide prospective cohort study	China	Patients with ICH; around 62% males and mean age of around 60 years	357	HbA1c categorized as <6.5% and ≥6.5%	12-month mortality HbA1c ≥ 6.5%: OR 1.08 (95% CI: 0.85, 1.37) 12-month stroke recurrence HbA1c ≥ 6.5%: OR 1.15 (95% CI: 0.90, 1.46) 12-month poor functional outcome HbA1c ≥ 6.5%: OR 1.93 (95% CI: 1.10, 3.38)
Zhang et al. (38)	Prospective registry study	China	Patients with spontaneous ICH; mean (SD) age of 59.8 (12.2) and 61% males	288	Continuous HbA1c (per unit increase)	Poor functional outcome at hospital discharge OR 1.28 (95% CI: 1.01, 1.33)
Liu et al. (39)	Prospective multicenter cohort study	China	Patients with spontaneous ICH; mean (SD) age of 59 years and 65% males	416	HbA1c categorized as <5.7%; 5.7–6.4% and ≥6.5%	12-month poor functional outcome HbA1c ≥ 6.5%: OR 2.35 (95% CI: 1.28, 4.29) HbA1c 5.7–6.4%: OR 1.11 (95% CI: 0.62, 2.00) 12-month mortality HbA1c ≥ 6.5%: OR 2.63 (95% CI: 1.34, 5.15) HbA1c 5.7–6.4%: OR 1.35 (95% CI: 0.64, 2.84)
Wang et al. (40)	Prospective cohort study	China	Patients with spontaneous ICH; mean (SD) age of 60 years and 40% females	150	HbA1c categorized as <5.7%; 5.7–6.4% and ≥6.5%	1-month poor functional outcome HbA1c ≥ 6.5%: OR 8.6 (95% CI: 1.77, 41.7) HbA1c 5.7–6.4%: OR 6.17 (95% CI: 1.40, 27.1)

The primary outcomes for this meta-analysis were mortality and functional dependence. The secondary outcomes were risk of symptomatic intracranial hemorrhage (sICH), early neurological complications, and stroke recurrence. Mortality was reported by studies as within 1 year of stroke, whereas functional outcomes were reported at or within 3 and 12 months from the onset of stroke. Symptomatic intracranial hemorrhage and early neurological deterioration/complications were reported by majority of studies within 24 h of stroke onset. Out of the five studies that reported sICH, four studied intracranial hemorrhage after recanalization therapies (20, 28, 31, 32), and one reported hemorrhagic transformation of the ischemic infarct independently of recanalization therapies (23).

The results of the quality evaluation of the included studies are provided in Supplementary Table 2. Overall, the quality of the included studies was judged to be good. Majority of studies reported on appropriate selection of participants, ascertainment of exposure and outcome, and had controlled for baseline differences in the cohorts.

### Findings for Acute Ischemic Stroke

#### HbA1c as Continuous

**Figure 2:**
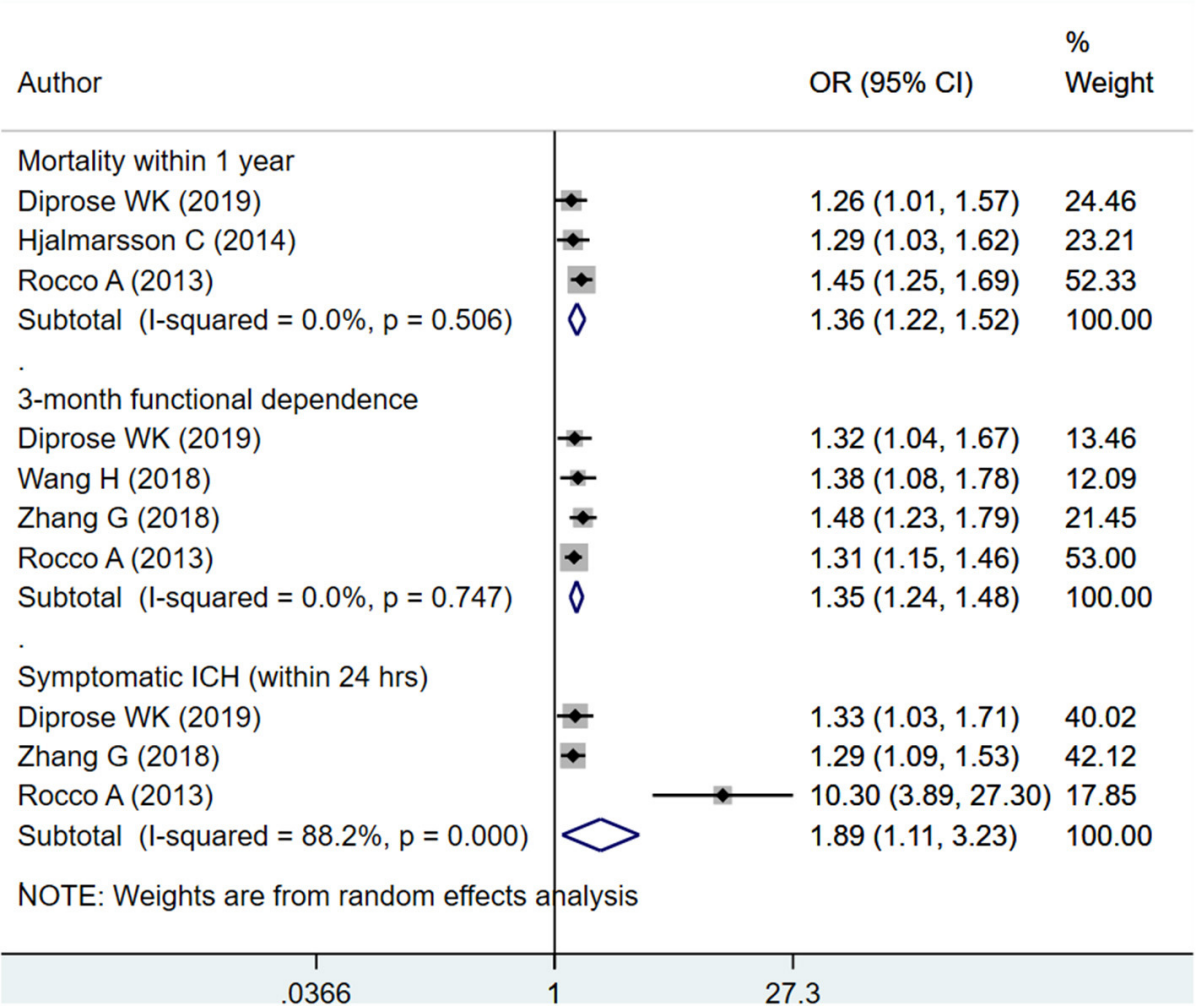
Pooled association of glycated hemoglobin (HbA1c) (continuous) with outcomes (mortality, functional dependence, and symptomatic intracranial hemorrhage) in patients with acute ischemic stroke.

Upon pooling of relevant studies, each unit increase in HbA1c was found to be associated with an increased risk of mortality within 1 year (OR 1.36; 95% CI: 1.22, 1.52; *I*^2^ = 0.0%; no. of studies, *N* = 3), increase risk of poor functional outcome or functional dependence at 3 months (OR 1.35; 95% CI: 1.24, 1.48; *I*^2^ = 0.0%; *N* = 4), and an increased risk of symptomatic intracranial hemorrhage (sICH) within 24 h of admission (OR 1.89; 95% CI: 1.11, 3.23; *I*^2^ = 88.2%; *N* = 3) (Figure 2). There was no evidence of publication bias using Egger's test, for any of the outcomes considered (*P* = 0.72 for mortality; *P* = 0.31 for poor functional outcome and *P* = 0.18 for sICH). Funnel plot is presented as Supplementary Figures 1–3.

#### HbA1c as Categorical

**Figure 3:**
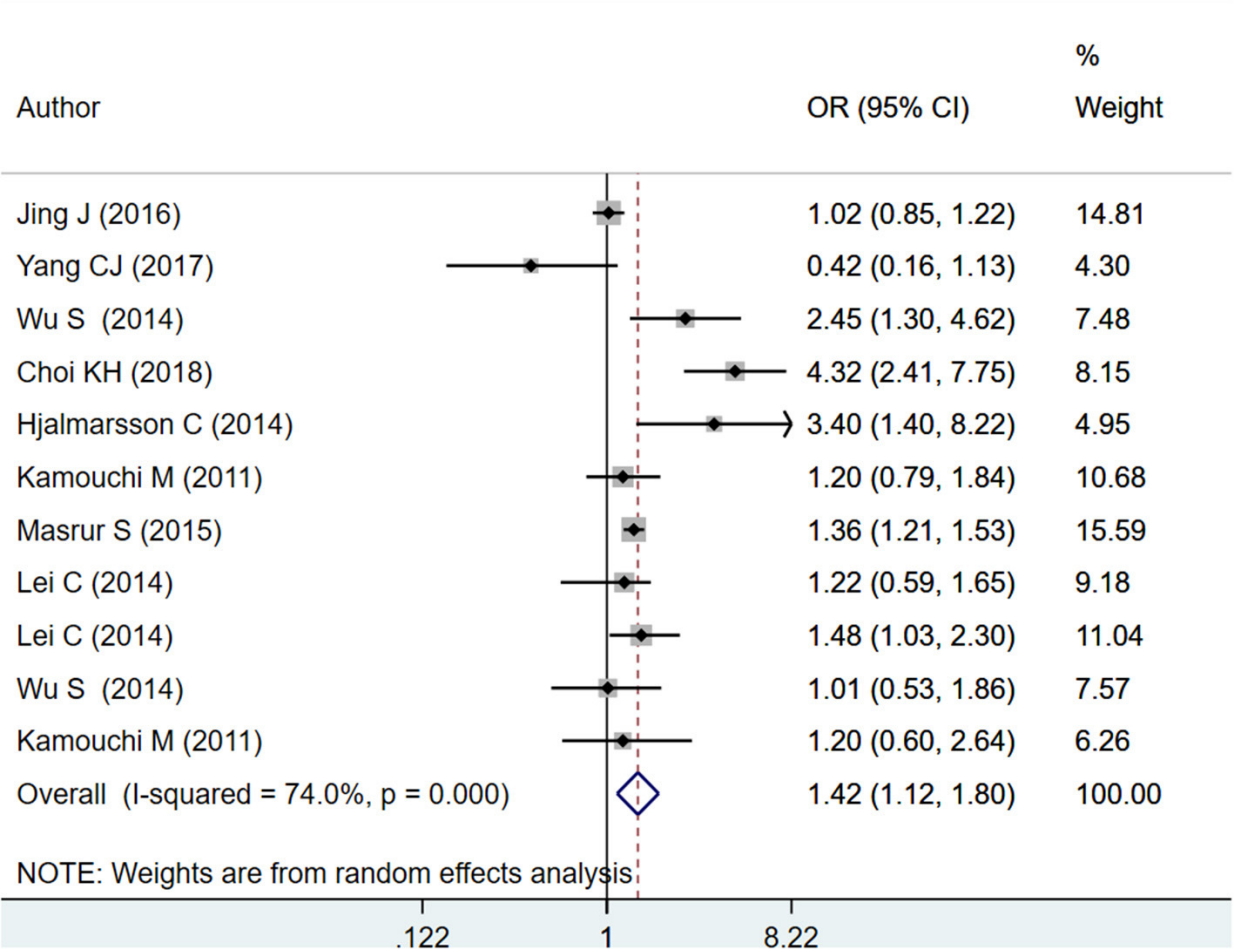
Pooled association of HbA1c ≥ 6.5% with mortality within 1 year of admission for acute ischemic stroke.

**Figure 4:**
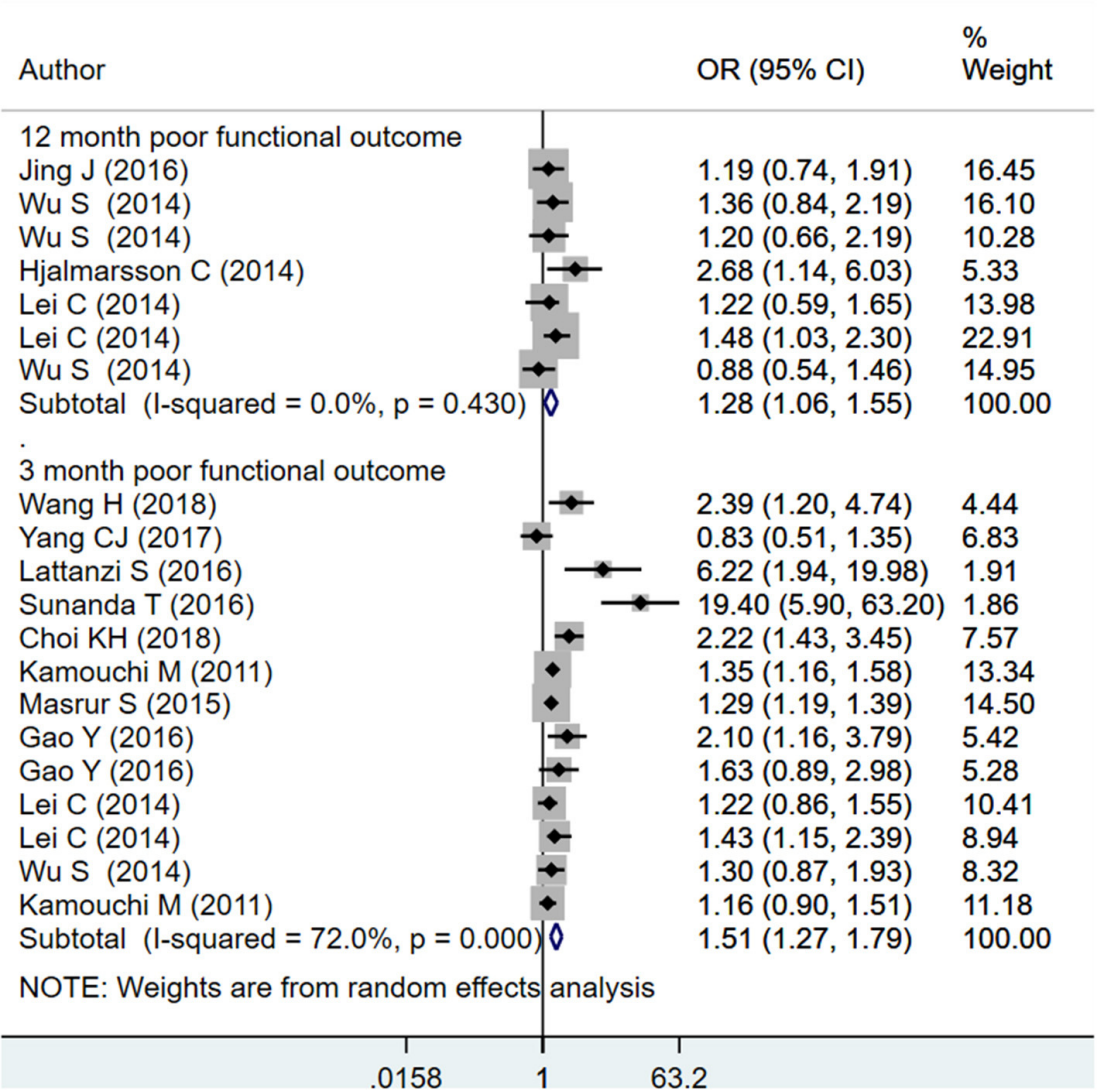
Pooled association of HbA1c ≥ 6.5% with poor functional outcomes at 3 and 12 months after the event of acute ischemic stroke.

The pooled effect sizes for HbA1c ≥ 6.5% indicate increased mortality within 1 year of admission for stroke (OR 1.42; 95% CI: 1.12, 1.80; *I*^2^ = 74.0%; *N* = 8) and increased risk of poor functional outcomes at 3 months (OR 1.51; 95% CI: 1.27, 1.79; *I*^2^ = 72.0%; *N* = 10) and 12 months (OR 1.28; 95% CI: 1.06, 1.55; *I*^2^ = 0.0%; *N* = 4) after the event of stroke (Figures 3, 4). On subgroup analysis based on the design of the studies, i.e., prospective or retrospective, a significant association was found between HbA1c ≥ 6.5%, and risk of mortality when studies that were prospective in design were pooled (OR 1.59; 95% CI: 1.04, 2.42; *I*^2^ = 81.9%; *N* = 5) but not when studies done retrospectively were pooled (OR 1.28; 95% CI: 0.89, 1.84; *I*^2^ = 60.3%; *N* = 4) (Supplementary Figure 4). Further, a significant association was found between HbA1c ≥ 6.5% and risk of poor functional outcome at both 3 and 12 months when studies that were prospective in design were pooled (at 3 months: OR 1.96; 95% CI: 1.29, 3.00; *I*^2^ = 80.0%; *N* = 5; at 12 months: OR 1.23; 95% CI: 1.01, 1.50; *I*^2^ = 0.0%; *N* = 3). Similar findings for risk of poor functional outcome were observed when studies that were retrospective in design were pooled (at 3 months: OR 1.33; 95% CI: 1.13, 1.56; *I*^2^ = 57.9%; *N* = 5; at 12 months: OR 2.68; 95% CI: 1.17, 6.16; *N* = 1) (Supplementary Figures 5, 6).

**Figure 5:**
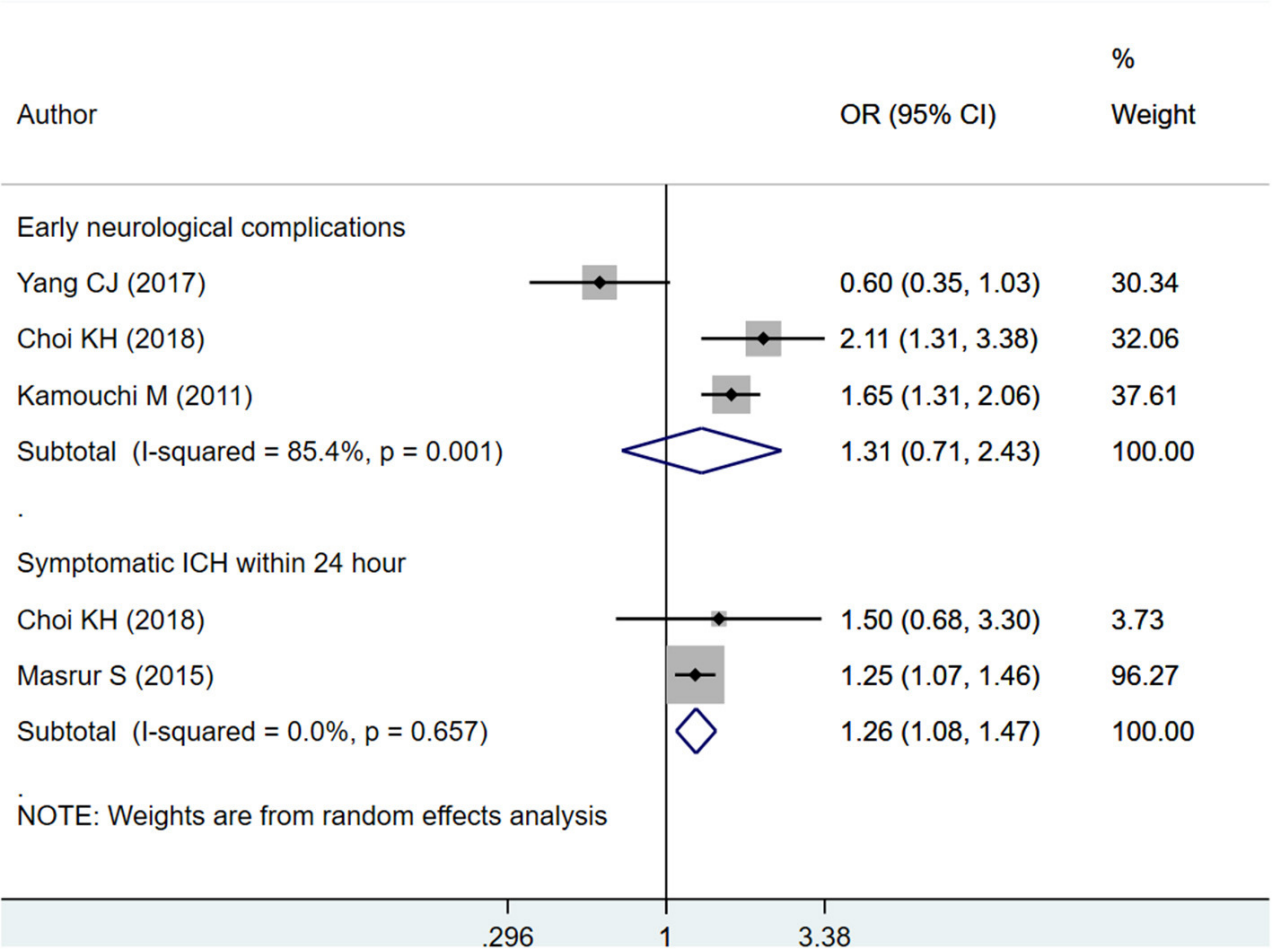
Pooled association of HbA1c ≥ 6.5% with early neurological complications and symptomatic intracranial hemorrhage (sICH) within 24 h of admission, in patients with acute ischemic stroke.

A significant association was also found between HbA1c ≥ 6.5% and risk of symptomatic intracranial hemorrhage (sICH) within 24 h of admission (OR 1.26; 95% CI: 1.08, 1.47; *I*^2^ = 0.0%; *N* = 2) (Figure 5). There was no signification association between high HbA1c values (i.e., HbA1c ≥ 6.5%) and risk of early neurological complications (OR 1.31; 95% CI: 0.71, 2.43; *I*^2^ = 85.4%; *N* = 3) (Figure 5). There was no evidence of publication bias using Egger's test, for any of the outcomes considered (*P* = 0.29 for mortality; *P* = 0.66 for poor functional outcome; *P* = 0.81 for sICH, and *P* = 0.54 for early neurological complications). Funnel plot is presented as Supplementary Figures 7–10.

On subgroup analysis, based on different values of HbA1c, there was no significant association with mortality within 1 year of admission for stroke (Supplementary Figure 11). This could be because of very few studies reporting association between subgroups based on different HbA1c values and mortality. The 3-month poor functional outcome was significantly associated with different subgroups based on HbA1c values (*HbA1c 5.9–6.7%:* OR 1.24; 95% CI: 1.01, 1.52, *N* = 3; *HbA1c 6.8–8.2%:* OR 1.24; 95% CI: 1.00, 1.53, *N* = 2; *HbA1c* > *8.2%:* OR 1.80; 95% CI: 1.13, 2.87, *N* = 2) (Supplementary Figures 12–14). Further, a dose–response relationship was observed with the magnitude of association being maximum in the subgroup with HbA1c >8.2%. Possibly due to very few studies that reported on 12-month functional outcome within subgroups based on HbA1c, the pooled association was non-significant.

### Findings for Hemorrhagic Stroke

#### HbA1c as Continuous

**Figure 6:**
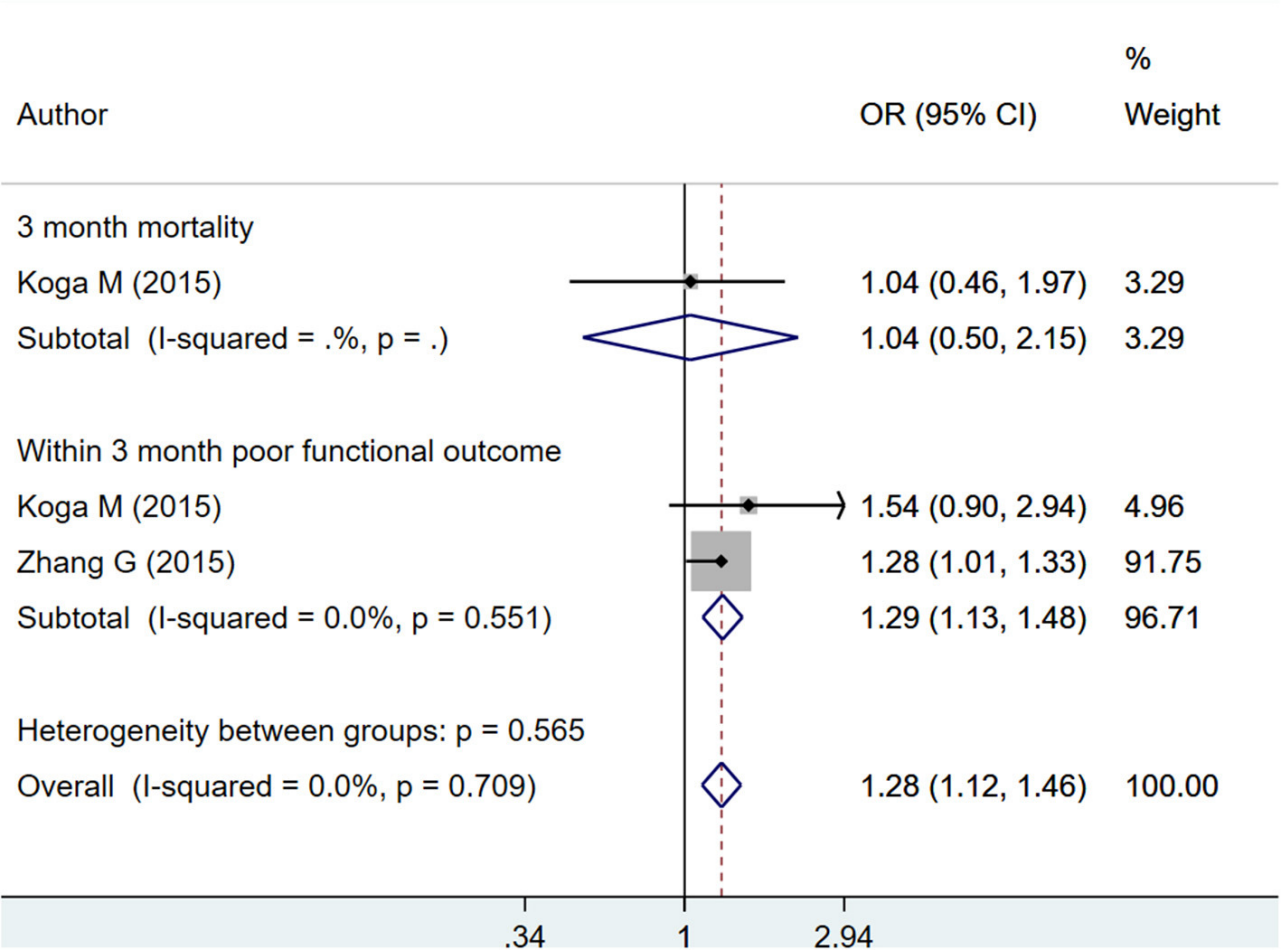
Pooled association of continuous HbA1c with mortality and poor functional outcomes at or within 3 months from time of admission for stroke, in patients with hemorrhagic stroke.

Each unit increase in HbA1c was found to be associated with increased risk of poor functional outcome within the first 3 months from the time of admission for stroke (OR 1.29; 95% CI: 1.13, 1.48; *I*^2^ = 0.0%; *N* = 2) (Figure 6). However, such significant association was not observed with 3-month mortality (OR 1.04; 95% CI: 0.50, 2.15; *N* = 1). The risk of “any” complication, i.e., either mortality or poor functional outcome, increased with each unit increase in HbA1c (OR 1.28; 95% CI: 1.12, 1.46; *I*^2^ = 0.0%; *N* = 2) (Figure 6). There was no evidence of publication bias using Egger's test, for any of the outcomes considered (*P* = 0.29 for mortality; *P* = 0.33 for poor functional outcome). Funnel plot is presented as Supplementary Figure 15.

#### HbA1c as Categorical

**Figure 7:**
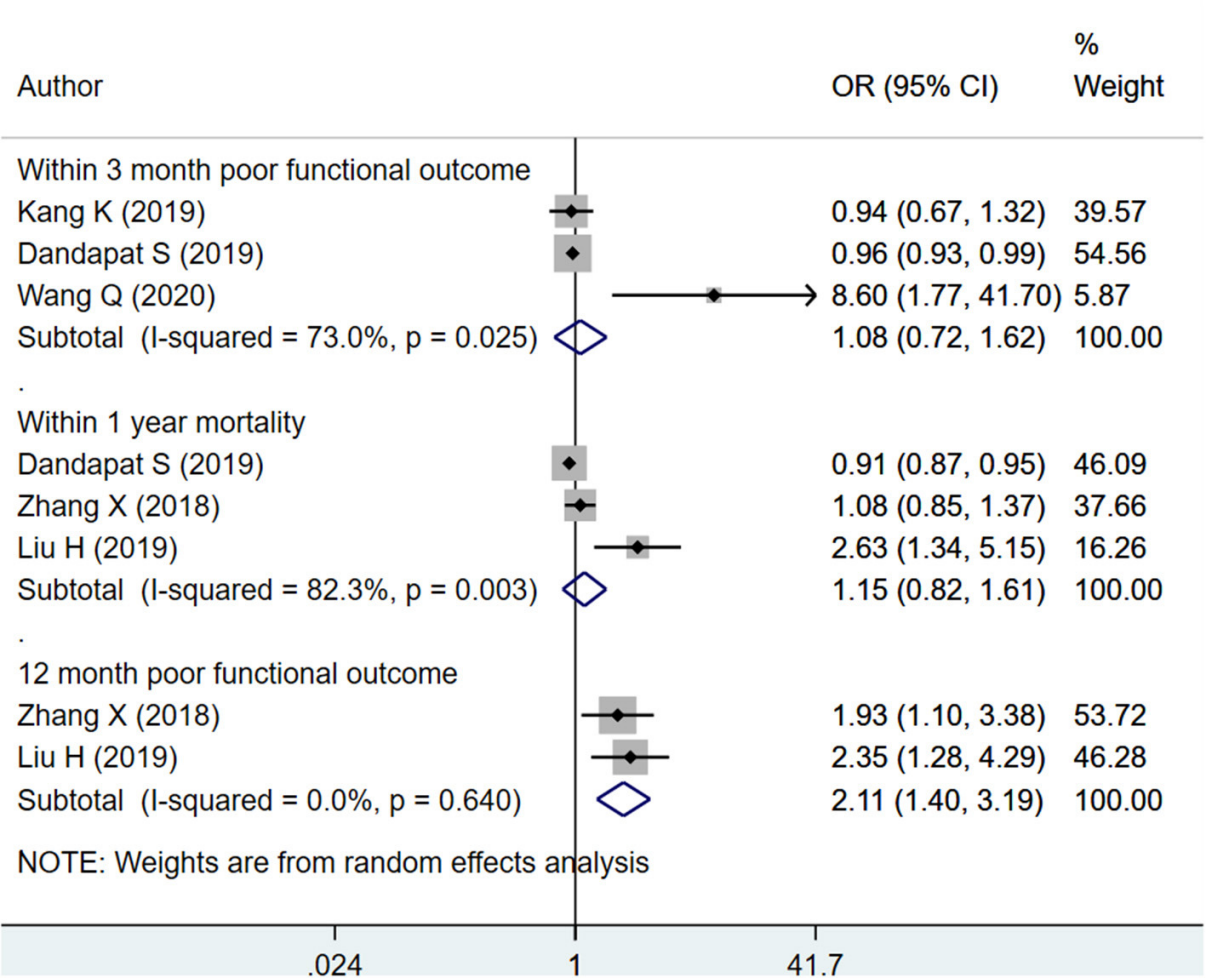
Pooled association of HbA1c ≥ 6.5% with functional outcomes and mortality in patients with hemorrhagic stroke.

The pooled effect sizes indicate that among patients with hemorrhagic stroke, HbA1c ≥ 6.5% is associated with increased risk of poor functional outcome at 12 months (OR 2.11; 95% CI: 1.40, 3.19; *I*^2^ = 0.0%; *N* = 2) but not poor functional outcome within 3 months (OR 1.08; 95% CI: 0.72, 1.62; *I*^2^ = 73.0%; *N* = 3) or mortality within 12 months (OR 1.15; 95% CI: 0.82, 1.61; *I*^2^ = 82.3%; *N* = 3) (Figure 7). There was no evidence of publication bias for any of the outcomes considered (*P* = 0.17 for mortality; *P* = 0.25 for poor functional outcomes). Funnel plot is presented as Supplementary Figure 16.

On subgroup analysis, there was no significant association with mortality within 1 year as well as functional outcomes at 3 and 12 months in subgroup with HbA1c between 5.7 and 6.4% (Supplementary Figure 17). In the subgroup of HbA1c between 6.0 and 7.9%, there was a significantly reduced risk of poor functional outcome within 3 months (OR 0.75; 95% CI: 0.56, 1.01; *I*^2^ = 0.0%; *N* = 2) and mortality within 12 months of stroke (OR 0.73; 95% CI: 0.62, 0.86; *N* = 1) (Supplementary Figure 18). Similarly, in the subgroup of HbA1c > 8.2%, although not statistically significant, there was a reduced risk of poor functional outcome within 3 months (OR 0.78; 95% CI: 0.57, 1.05; *I*^2^ = 0.0%; *N* = 2) (Supplementary Figure 19). The risk of mortality with 12 months of stroke (OR 0.78; 95% CI: 0.65, 0.93; *N* = 1) was significantly reduced (Supplementary Figure 19).

## Discussion

Glycated hemoglobin (HbA1c) has been shown to be associated with increased risk of first-onset stroke (41). A systematic review of 29 articles and around 500,000 participants showed that compared with HbA1c of <5.7%, HbA1c of ≥6.5% was associated with an increased risk of first-ever stroke (hazard ratio 2.15; 95% CI: 1.76, 2.63). The review also documented that for every 1% increment in HbA1c, there was a higher associated risk of first-ever ischemic stroke (hazard ratio 1.49; 95% CI: 1.32, 1.69) (41). So, while we understand well that increase in HbA1c is associated with increased risk of stroke, we do not understand the strength and nature of association of HbA1c with outcomes of stroke. The current meta-analysis was conducted with the intent to examine the association, if any, between HbA1c values and outcomes in patients with ischemic and hemorrhagic stroke. The findings indicate that each unit increase in HbA1c (continuous) and HbA1c ≥ 6.5% was found to be associated with an increased risk of mortality within 1 year, increased risk of poor functional outcome, and increased risk of symptomatic intracranial hemorrhage (sICH) in patients with ischemic stroke. In patients with hemorrhagic stroke, each unit increase in HbA1c was found to be associated with increased risk of poor functional outcome, but no significant association was observed with mortality. Similarly, HbA1c ≥ 6.5% was associated with increased risk of poor functional outcome but not with mortality. These findings indicate that glycated hemoglobin could serve as a useful marker to predict the outcomes in patients with stroke and consequently, the required management could be instituted.

Most of the included studies were consistent and showed high HbA1c to be associated with poor outcomes, yet there were few studies that reported findings in the opposite direction, particularly in hemorrhagic stroke patients. The findings of our subgroup analysis also show similar patterns. We did a subgroup analysis based on different cutoffs for HbA1c. Among those with ischemic stroke, no significant association was noted for any of the HbA1c categories with mortality within 1 year of admission. This might be because of very few studies reporting this association. Nonetheless, there was a clear dose–response relationship between different subgroups based on HbA1c values (*HbA1c 5.9–6.7%*; *HbA1c 6.8–8.2%*; *HbA1c* > *8.2%*) and short-term poor functional outcomes (within 3 months of admission for stroke). The magnitude of association was maximum in the subgroup with HbA1c > 8.2%. In those with hemorrhagic stroke, contrary to the current belief that higher HbA1c values will be associated with poor outcomes, in the subgroup of HbA1c between 6.0 and 7.9%, there was a significantly reduced risk of poor functional outcome within 3 months and mortality within 12 months of stroke. Similarly, in the subgroup of HbA1c > 8.2%, there was a reduced risk of poor functional outcome within 3 months, and the risk of mortality with 12 months of stroke was also significantly reduced. Studies have indicated low HbA1c to be associated with liver disease, low fibrinogen, and anemia, all of which could be expected to raise the risk of bleeding and increased hematoma volume (35, 42, 43). With high HbA1c levels, these might be averted, and this might explain the observed paradox.

Levels of blood glucose at the time of admission for stroke has been shown to have a positive association with the levels of HbA1c (44–46). The effect of uncontrolled blood glucose on infarct size and severity of the stroke is thought to be mediated through triggering of inflammatory pathways (44, 46). A poor glycemic status before and in the hyperacute stage of the stroke can therefore lead to worsening of the ischemic damage and consequent poor recovery. The high concentrations of HbA1c may also be an expression of unattention to a healthy lifestyle and poor adherence to treatment for coexisting vascular risk factors and related medical conditions. All these put together can have a detrimental impact on the outcomes of patients. The findings of the study serve as a useful evidence to support clinical programs aimed at better glycemic control in patients with diabetes as adequate pre-stroke glycemic control was found to decrease the risk of unfavorable outcomes. The findings also indicate that measurement of HbA1c could be a good addition to the decision support tools for endovascular thrombectomy; however, the efficacy of this approach needs further evaluation.

As discussed above, a major thrust of the current treatment practice is to focus on intensive and tight glucose control at the time of admission for a stroke event. While that is important, care must be instituted that an event of hypoglycemia does not ensue as this might lead to poor clinical outcomes. Apart from hyperglycemia and hypoglycemia, there is another important, yet overlooked, form of dysglycemia known as glycemic variability (GV) (47). It denotes the degree of fluctuation in the glucose levels over a period of time. Empirical studies have shown GV to be associated with poor functional outcomes, particularly in patients with acute ischemic stroke. However, it must be acknowledged that currently, there is no harmonized and universally accepted index to express GV, and until the time, more data are available on the relationship between GV and outcomes in stroke patients. It is preferred that continuous glucose monitoring is included in the management protocol for stroke (47).

There were some limitations of the study. Studies had used different cutoffs for categorizing HbA1c, and that posed difficulty in performing the analysis, particularly the subgroup analysis. Further, the timing of measurement of HbA1c varied between different studies and that could also have contributed to the heterogeneity observed in the meta-analysis. For some of the outcomes, such as 12-month functional outcome within subgroups based on HbA1c for patients with ischemic stroke, there were very limited number of studies (as low as one in number) that curtailed appropriate pooling of findings and also led to non-significant pooled estimates. Adjusted odd's ratios, as presented in the included studies, were pooled; the variables adjusted in the model may be different for different studies, and that may contribute to the heterogeneity in the pooled findings. Also, quite a few studies included in the analysis had a retrospective design, which may have led to selection bias.

HbA1c is clinically easy to measure, reflects long-term glycemic control, and is unaffected by transient changes in blood glucose levels. The findings of the meta-analysis provide evidence that HbA1c could be used as a marker to predict poor outcomes in patients with ischemic or hemorrhagic stroke. The findings call for regular monitoring and routine HbA1c testing at admission.

## Data Availability Statement

The original contributions presented in the study are included in the article/Supplementary Material, further inquiries can be directed to the corresponding author/s.

## Author Contributions

YB conceived and designed the study and wrote the paper. YB and DG were involved in literature search, data collection, and analyzed the data. DG reviewed and edited the manuscript. Both authors read and approved the final manuscript.

## Conflict of Interest

The authors declare that the research was conducted in the absence of any commercial or financial relationships that could be construed as a potential conflict of interest.

## Supplementary Material

Supplementary Figure 1 Funnel plot for publication bias with respect to HbA1c (continuous) and mortality within 1 year of admission for acute ischemic stroke.

Supplementary Figure 2 Funnel plot for publication bias with respect to HbA1c (continuous) and functional outcomes in patients with acute ischemic stroke.

Supplementary Figure 3 Funnel plot for publication bias with respect to HbA1c (continuous) and symptomatic intracranial hemorrhage in patients with acute ischemic stroke.

Supplementary Figure 4 Subgroup analysis for mortality based on prospective/retrospective studies.

Supplementary Figure 5 Subgroup analysis for functional outcome based on prospective/retrospective studies (at 3 months).

Supplementary Figure 6 Subgroup analysis for functional outcome based on prospective/retrospective studies (at 12 months).

Supplementary Figure 7 Funnel plot for publication bias with respect to HbA1c (categorical; ≥6.5%) and mortality within 1 year in patients with acute ischemic stroke.

Supplementary Figure 8 Funnel plot for publication bias with respect to HbA1c (categorical; ≥6.5%) and functional outcomes in patients with acute ischemic stroke.

Supplementary Figure 9 Funnel plot for publication bias with respect to HbA1c (categorical; ≥6.5%) and symptomatic intracerebral hemorrhage in patients with acute ischemic stroke.

Supplementary Figure 10 Funnel plot for publication bias with respect to HbA1c (categorical; ≥6.5%) and early neurological deterioration in patients with acute ischemic stroke.

Supplementary Figure 11 Forest plot with respect to subgroups based on different HbA1c categories and mortality within 1 year in patients with acute ischemic stroke.

Supplementary Figure 12 Pooled association between HbA1c value ranging from 5.9 to 6.7% with functional outcomes at 3 and 12 months after acute ischemic stroke.

Supplementary Figure 13 Pooled association between HbA1c value ranging from 6.8 to 8.2% with functional outcomes at 3 and 12 months after acute ischemic stroke.

Supplementary Figure 14 Pooled association between HbA1c value > 8.2% with functional outcomes at 3 and 12 months after acute ischemic stroke.

Supplementary Figure 15 Funnel plot for publication bias with respect to HbA1c (continuous) and mortality as well functional outcomes at or within 3 months from time of admission for stroke, in patients with hemorrhagic stroke.

Supplementary Figure 16 Funnel plot for publication bias with respect to HbA1c ≥ 6.5% and mortality within 12 months as well functional outcomes within 3 months or at 12 months from time of admission for stroke, in patients with hemorrhagic stroke.

Supplementary Figure 17 Pooled association between HbA1c value ranging from 5.7 to 6.4% with mortality and functional outcomes, in patients with hemorrhagic stroke.

Supplementary Figure 18 Pooled association between HbA1c value ranging from 6.0 to 7.9% with mortality and functional outcomes, in patients with hemorrhagic stroke.

Supplementary Figure 19 Pooled association between HbA1c value > 8.2% with mortality and functional outcomes, in patients with hemorrhagic stroke.

Supplementary Table 1 Search strategy for identification of studies to be included in the review.

Supplementary Table 2 Author's judgements about study quality using the adapted Ottawa-Newcastle Risk of Bias Assessment tool.
