# Supplementary material for: Glycated Hemoglobin as a Marker for Predicting Outcomes of Patients With Stroke (Ischemic and Hemorrhagic): A Systematic Review and Meta-Analysis
Source: Front Neurol. 2021 Mar 31;12:642899. doi: 10.3389/fneur.2021.642899 (PMC8044393; doi:10.3389/fneur.2021.642899)

Supplementary figure 1

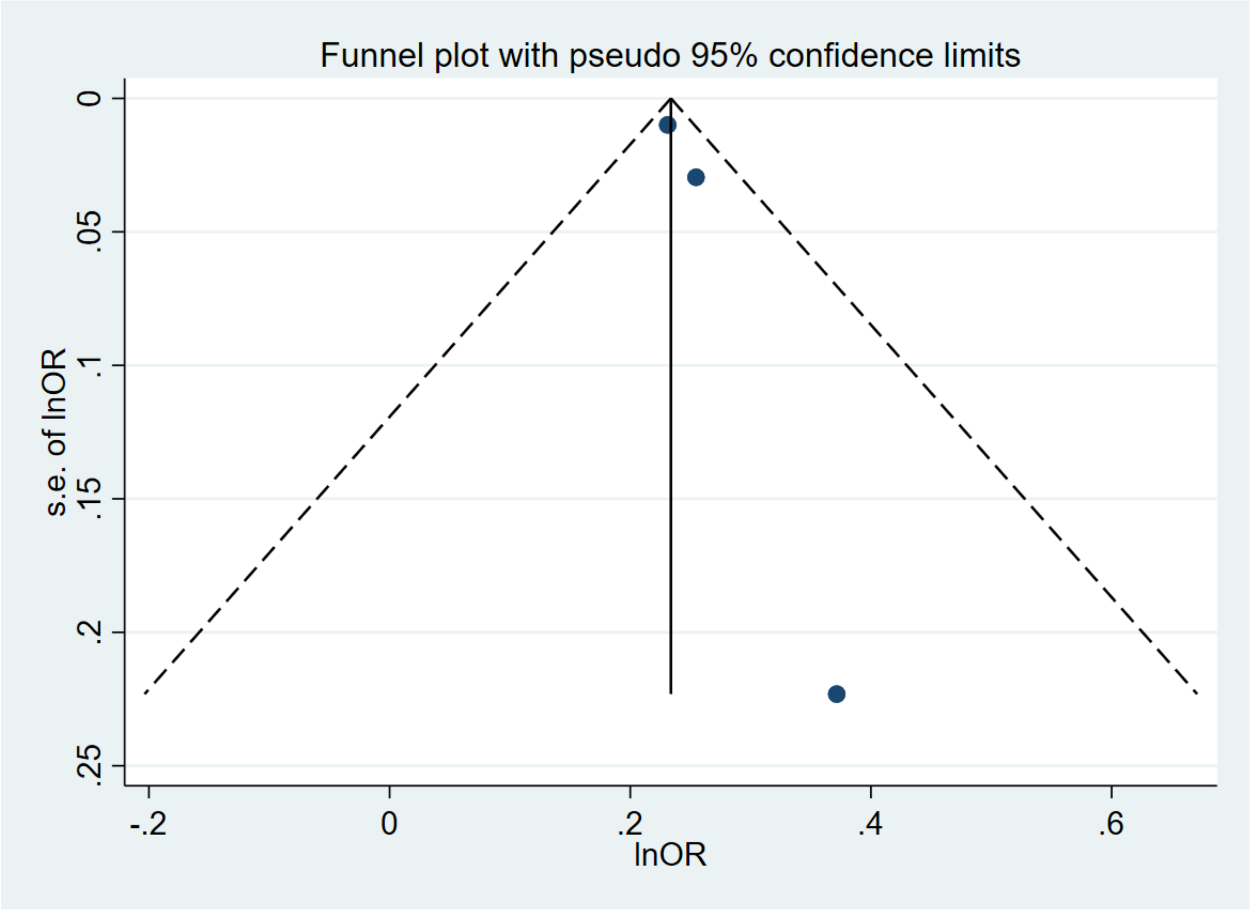

Supplementary figure 2

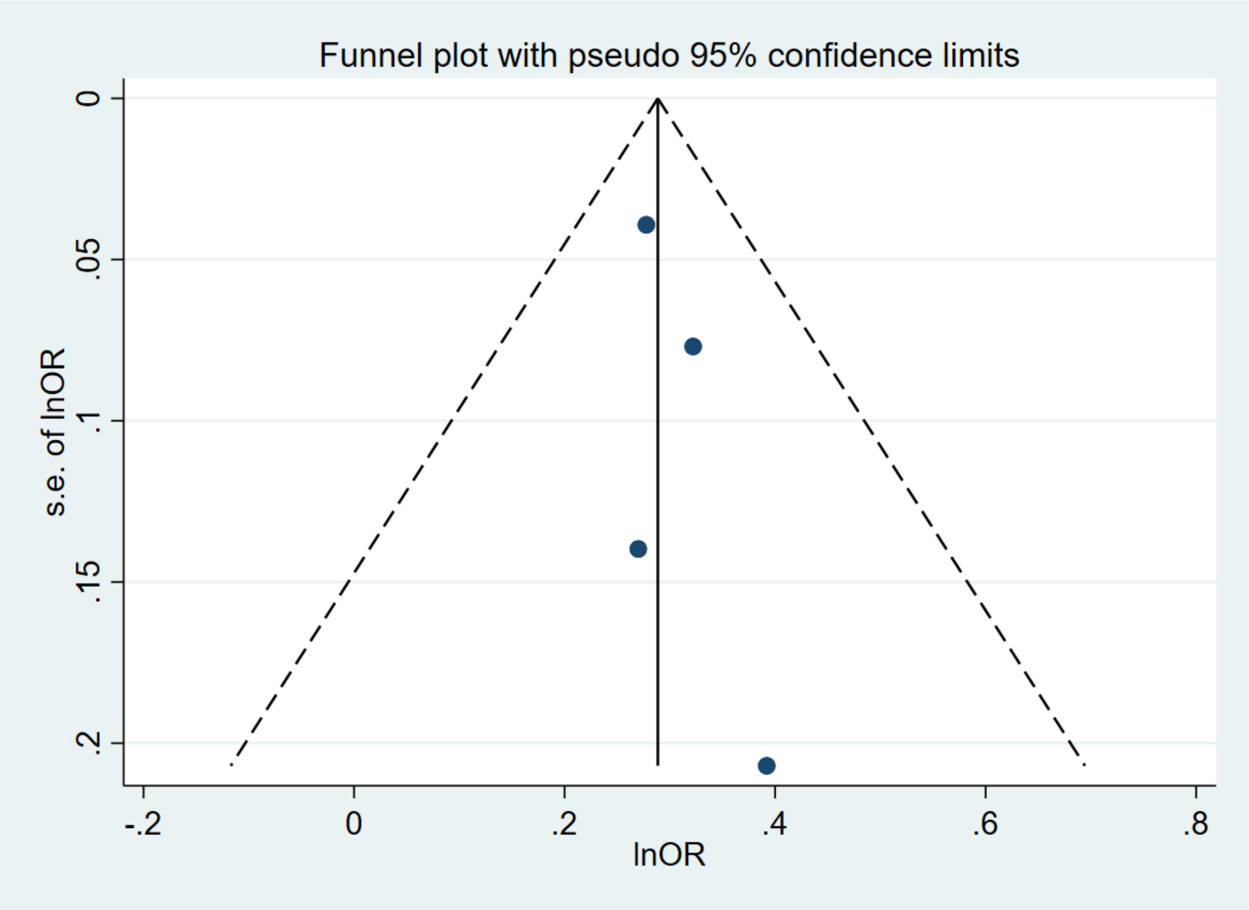

Supplementary figure 3

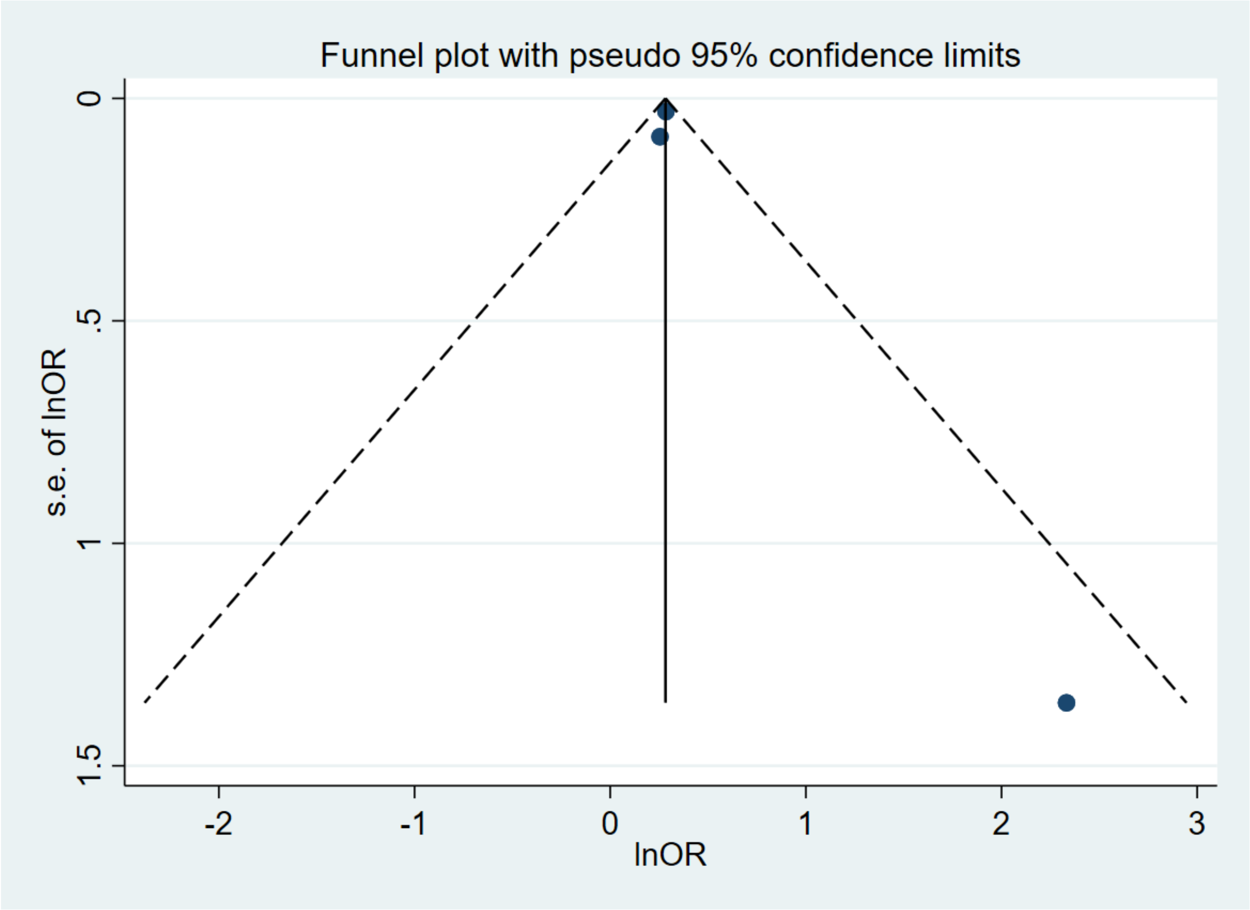

Supplementary figure 4

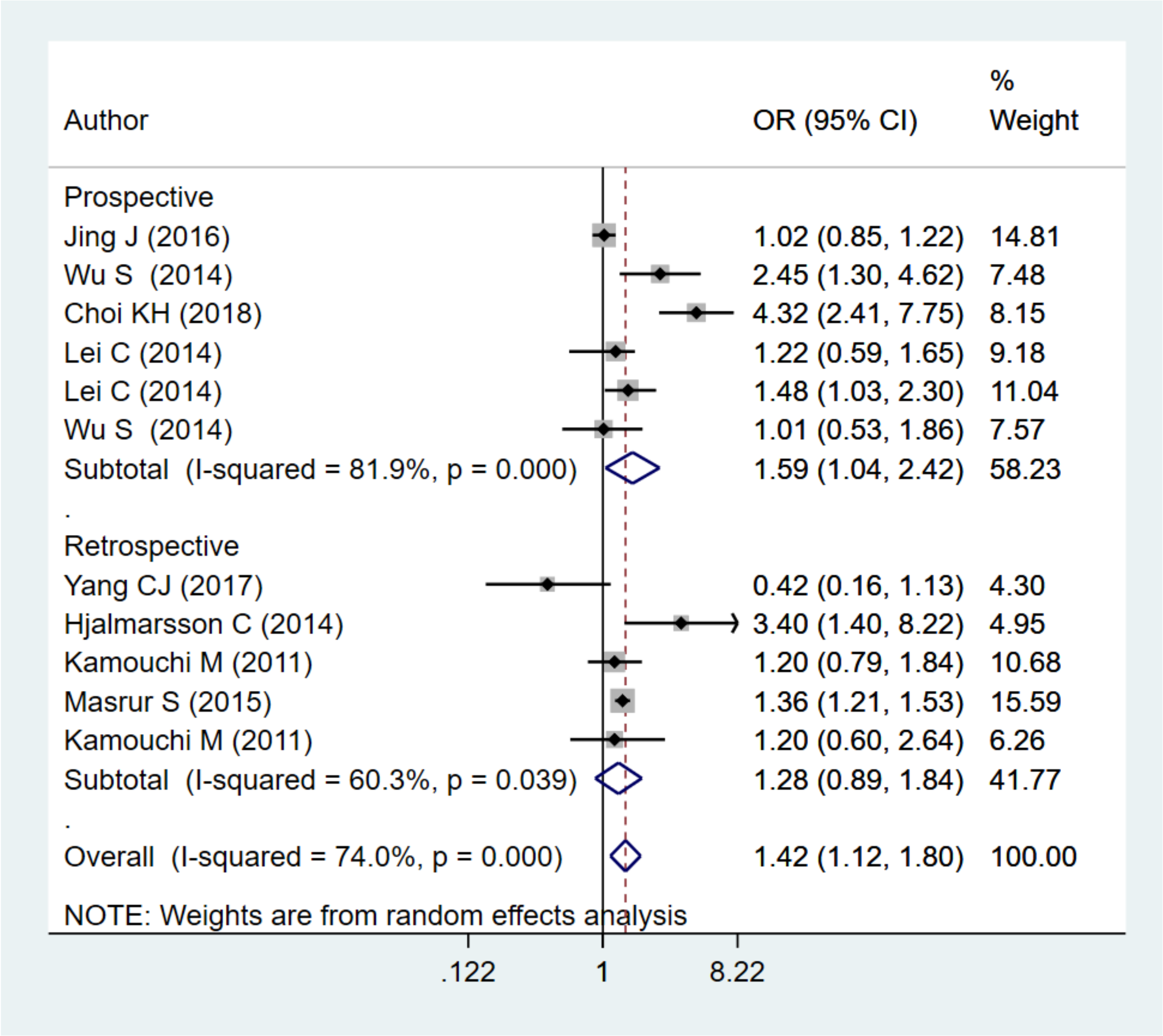

Supplementary figure 5

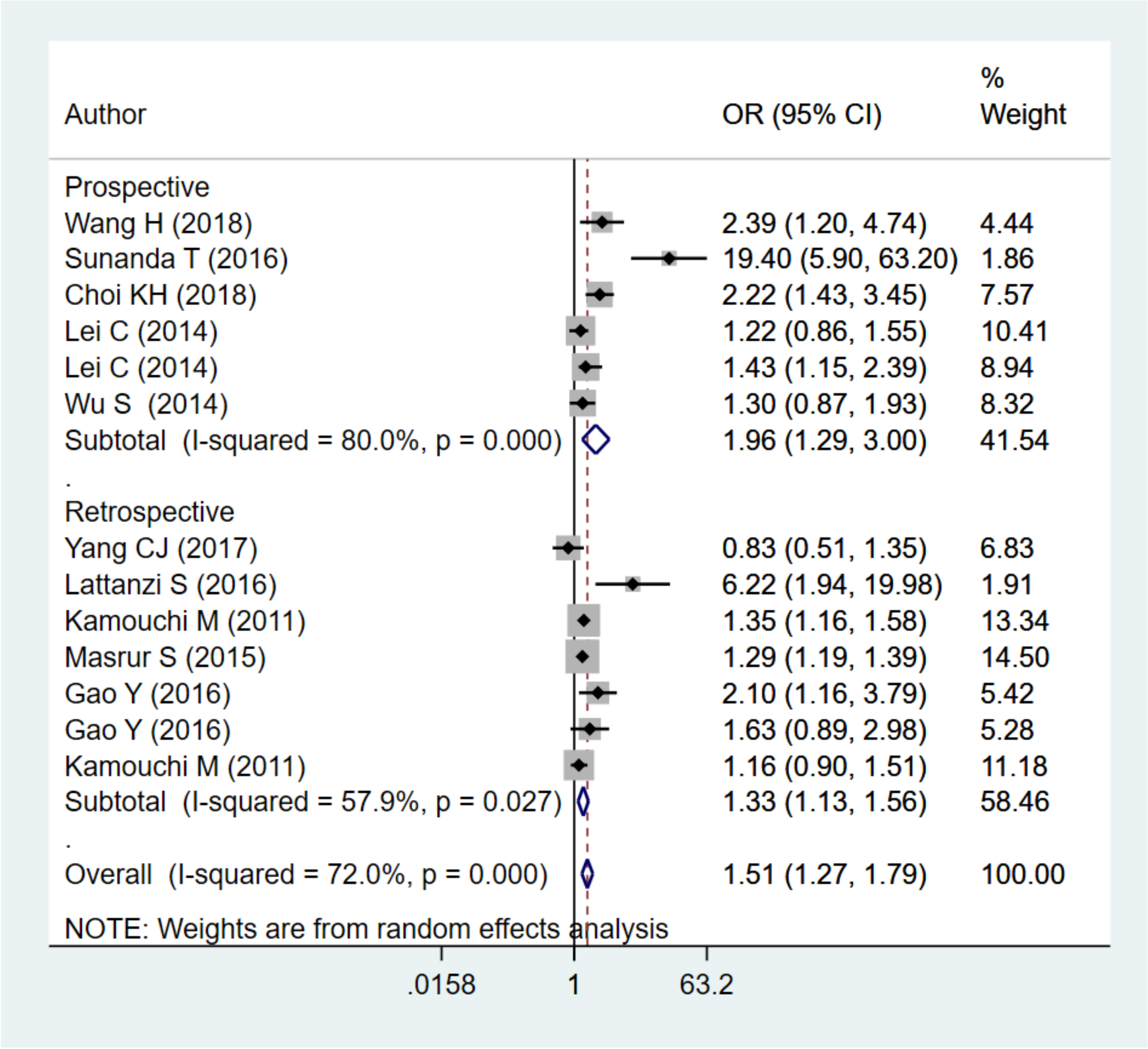

Supplementary figure 6

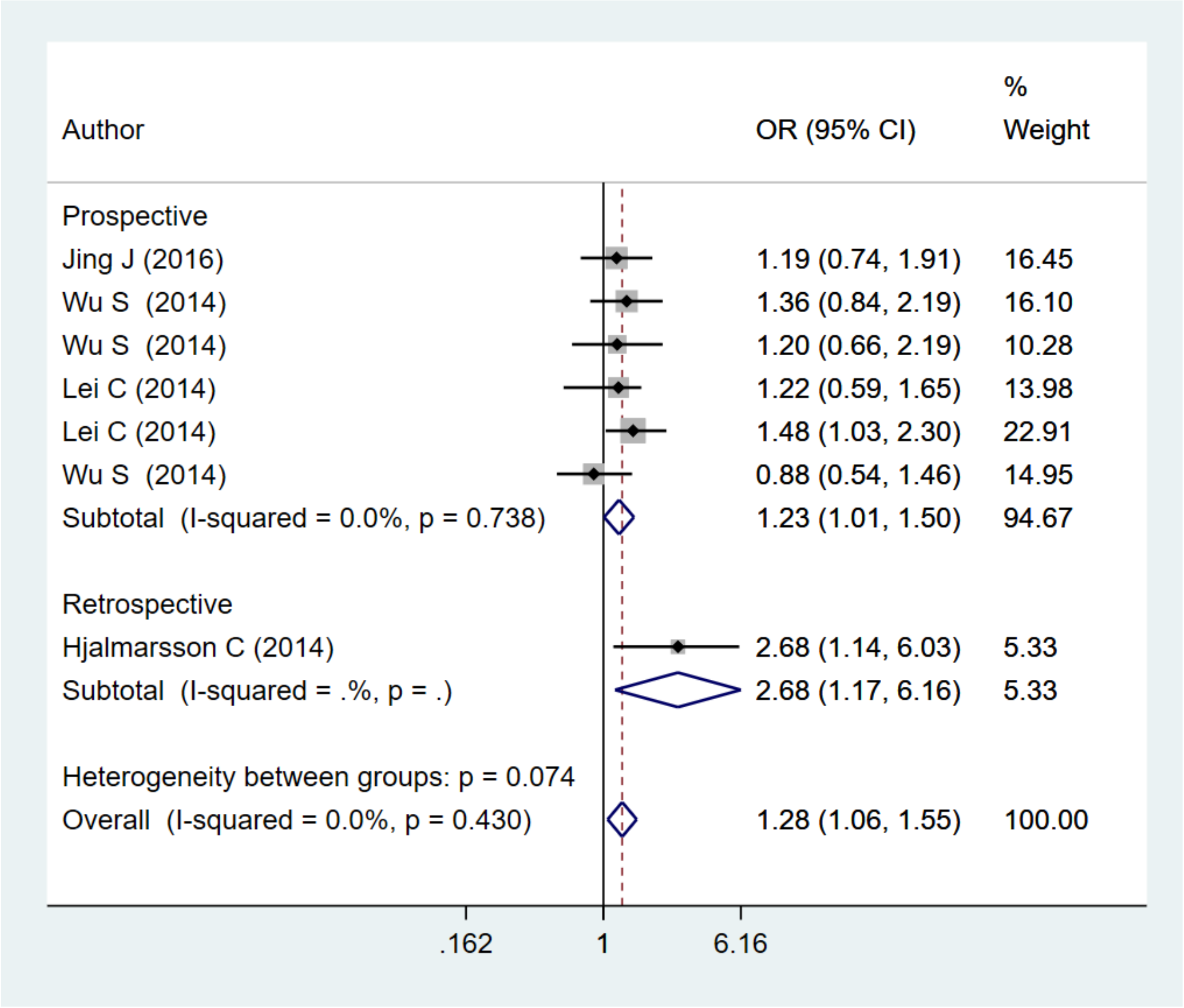

Supplementary figure 7

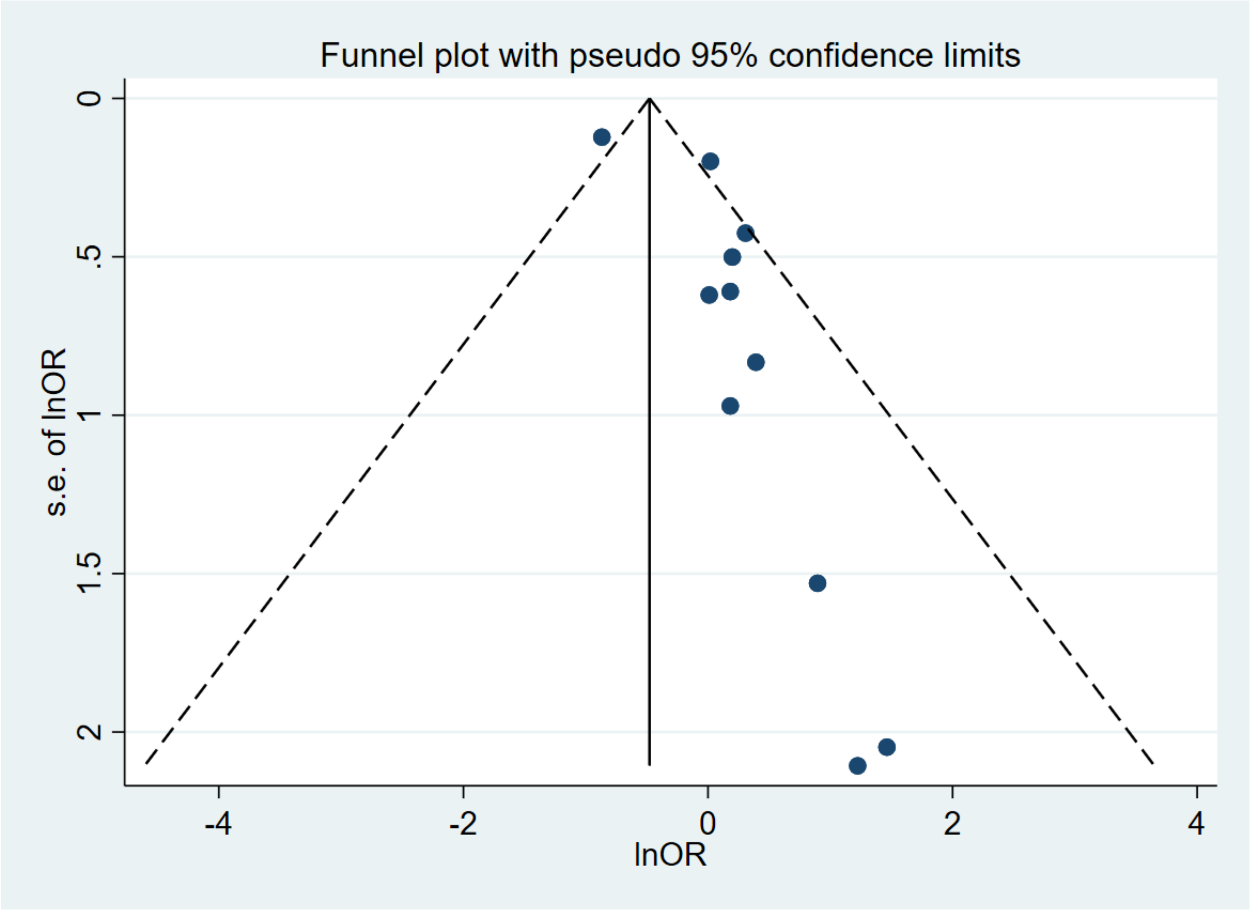

Supplementary figure 8

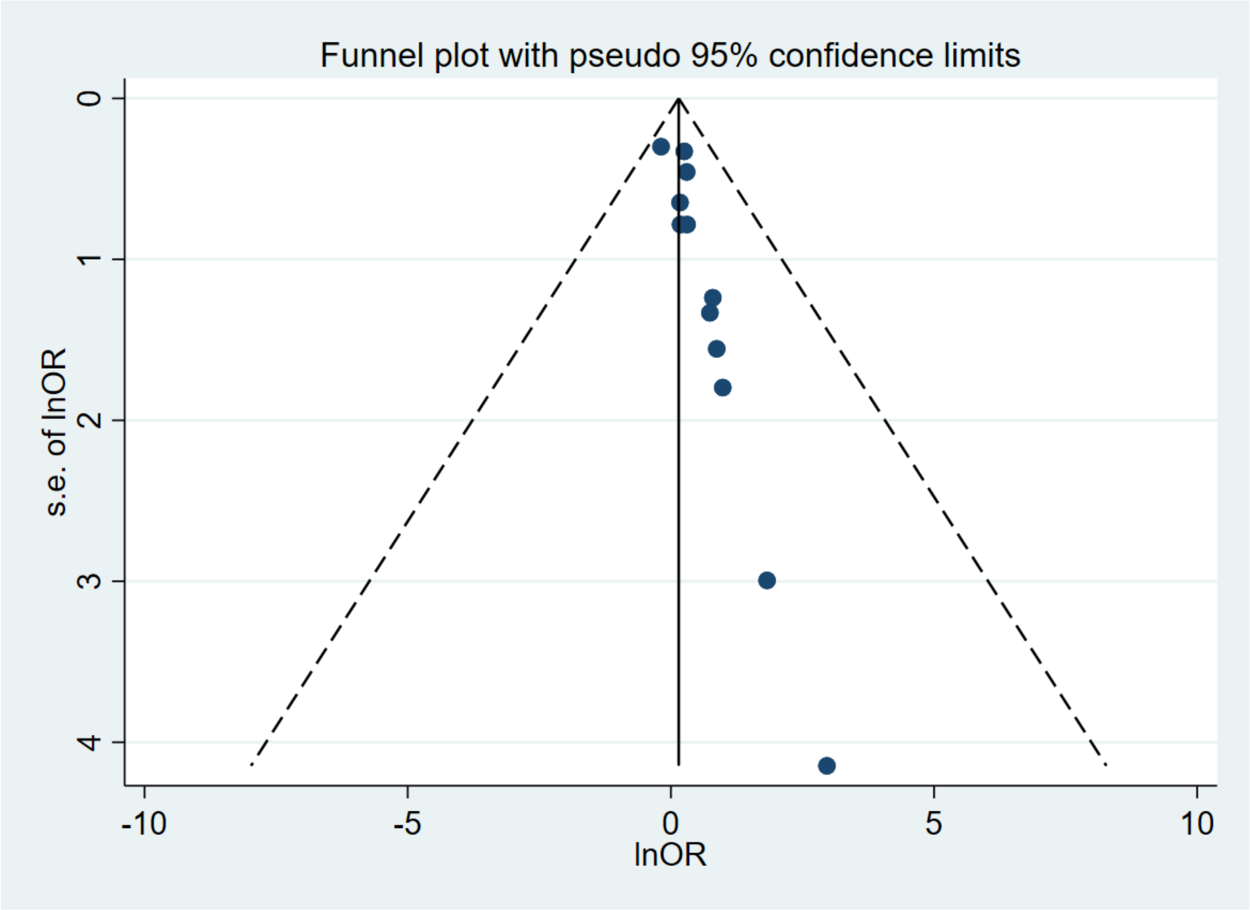

Supplementary figure 9

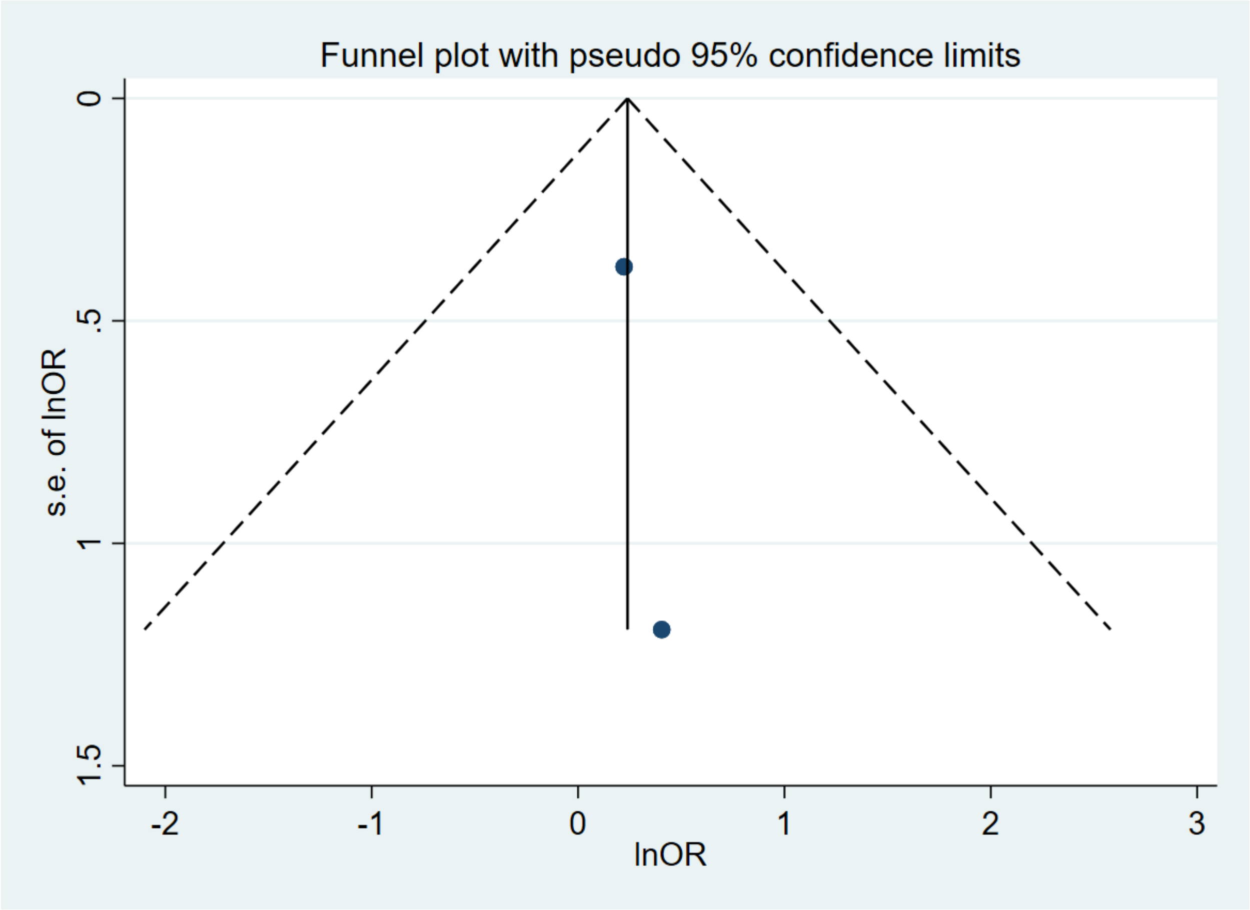

Supplementary figure 10

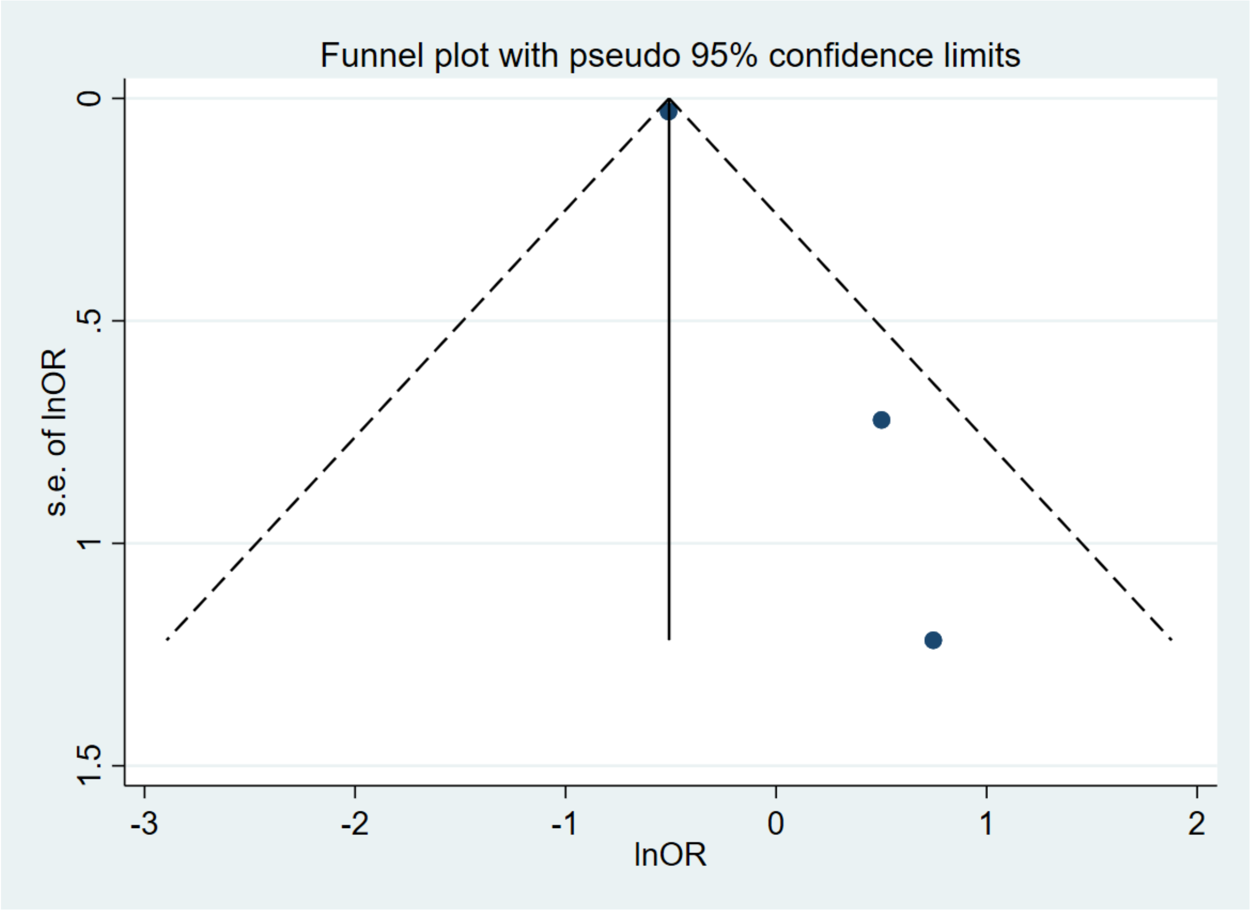

Supplementary figure 11

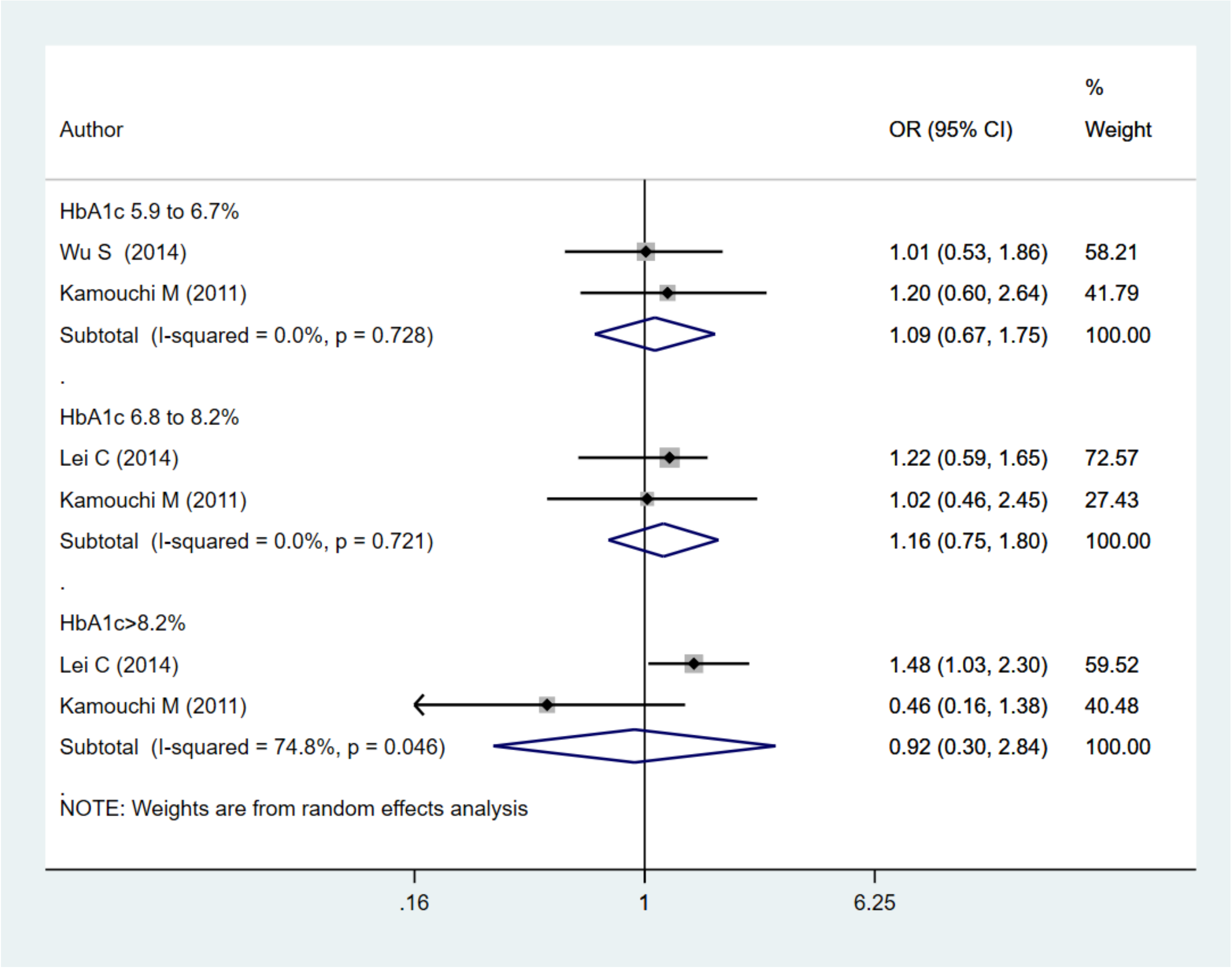

Supplementary figure 12

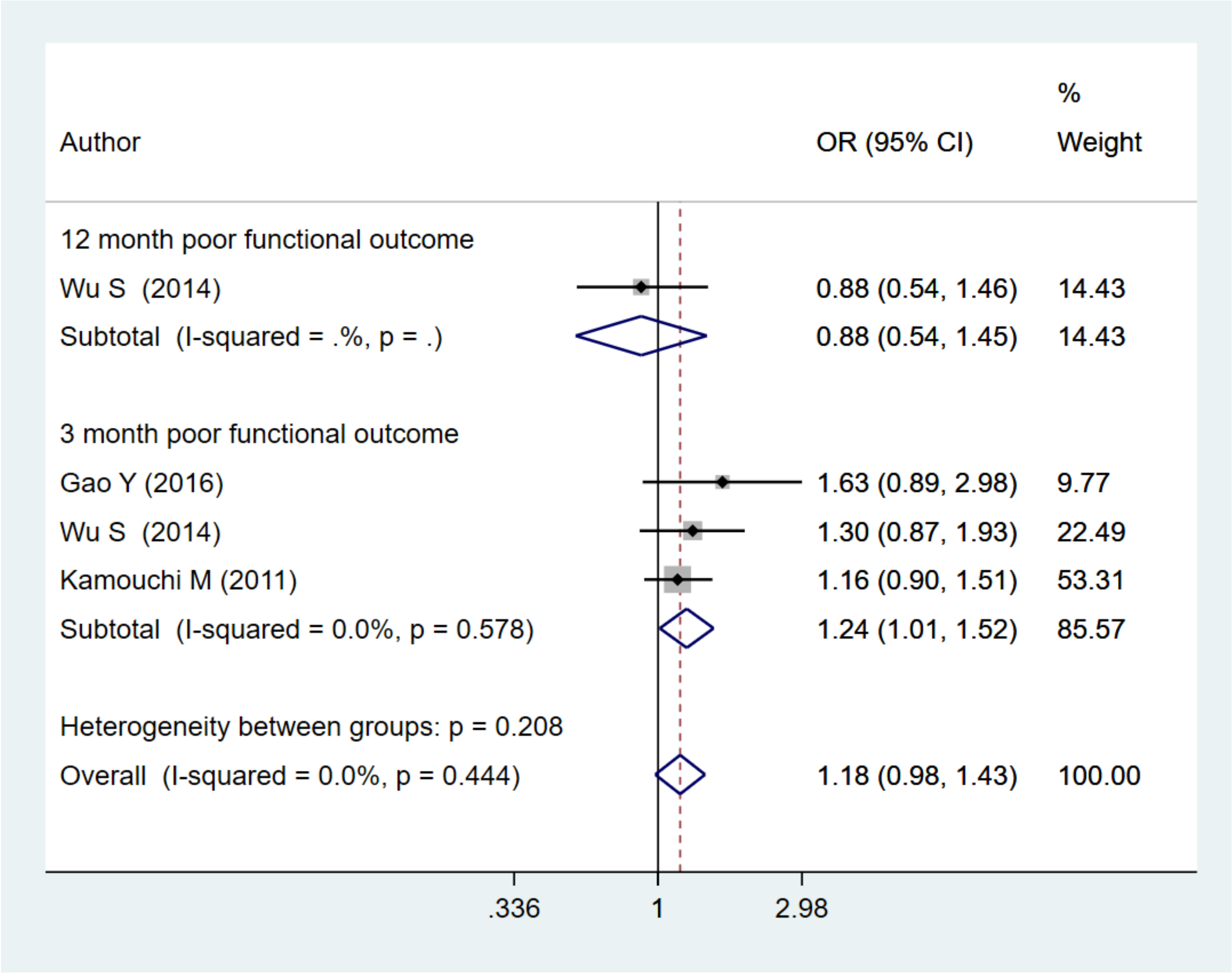

Supplementary figure 13

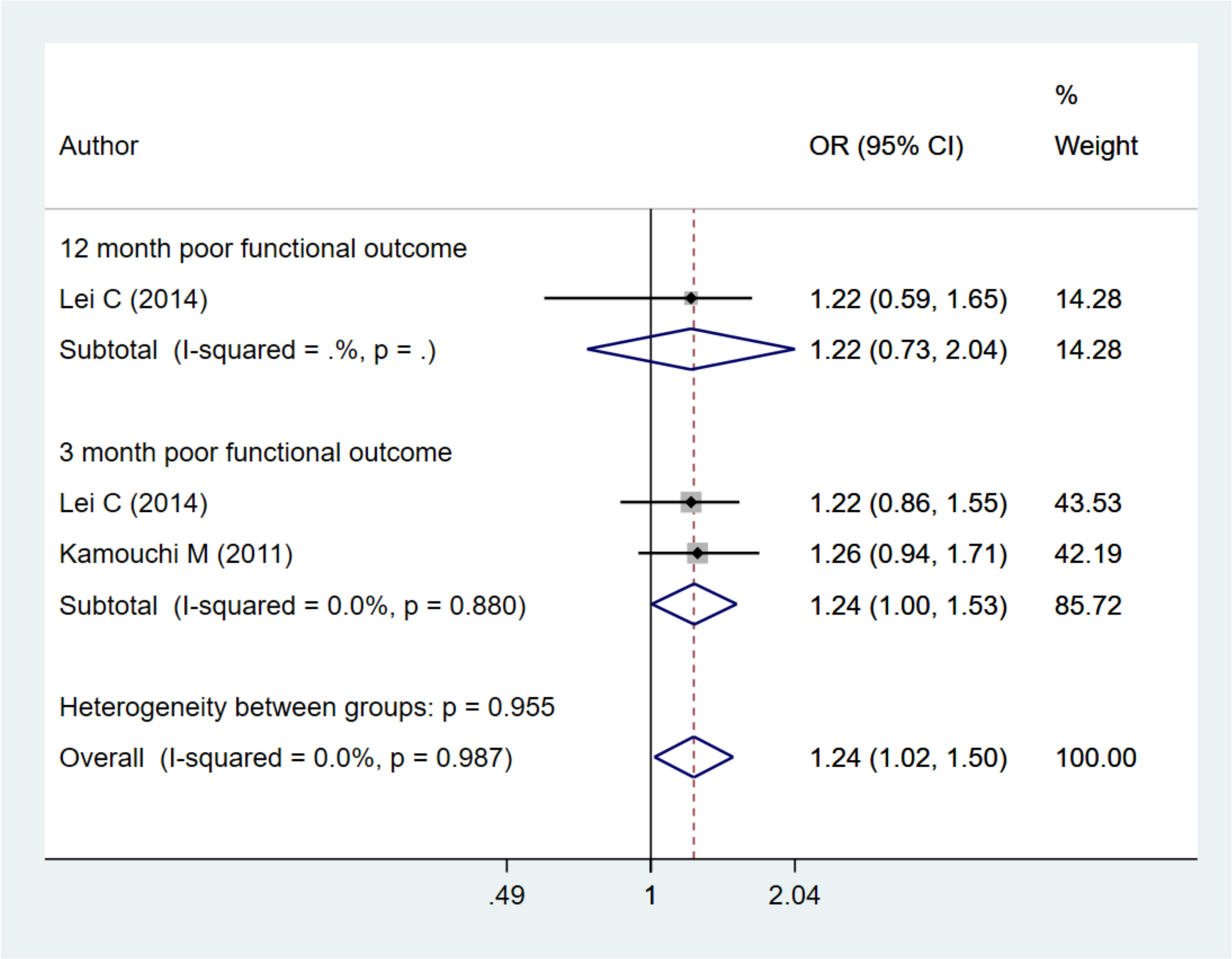

Supplementary figure 14

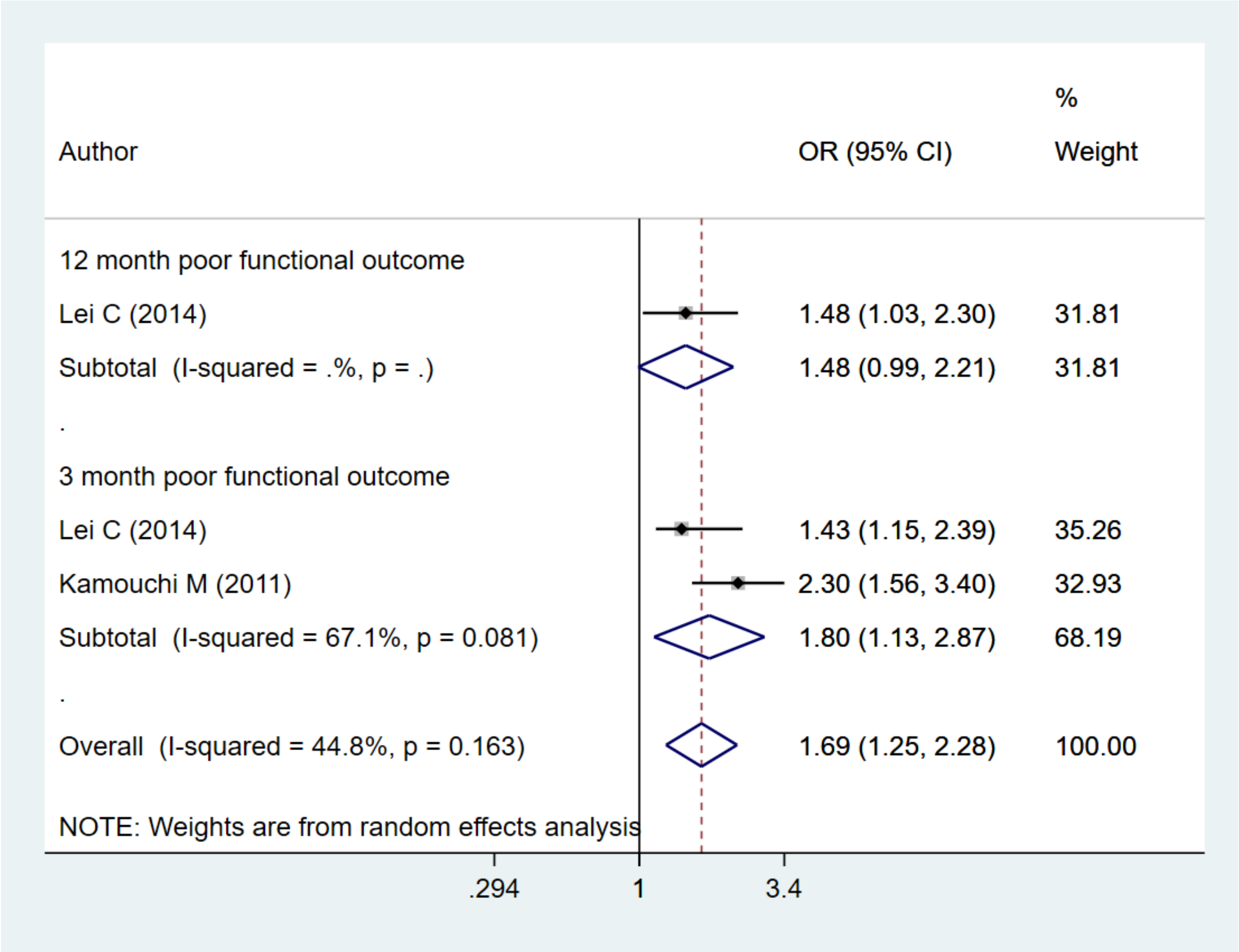

Supplementary figure 15

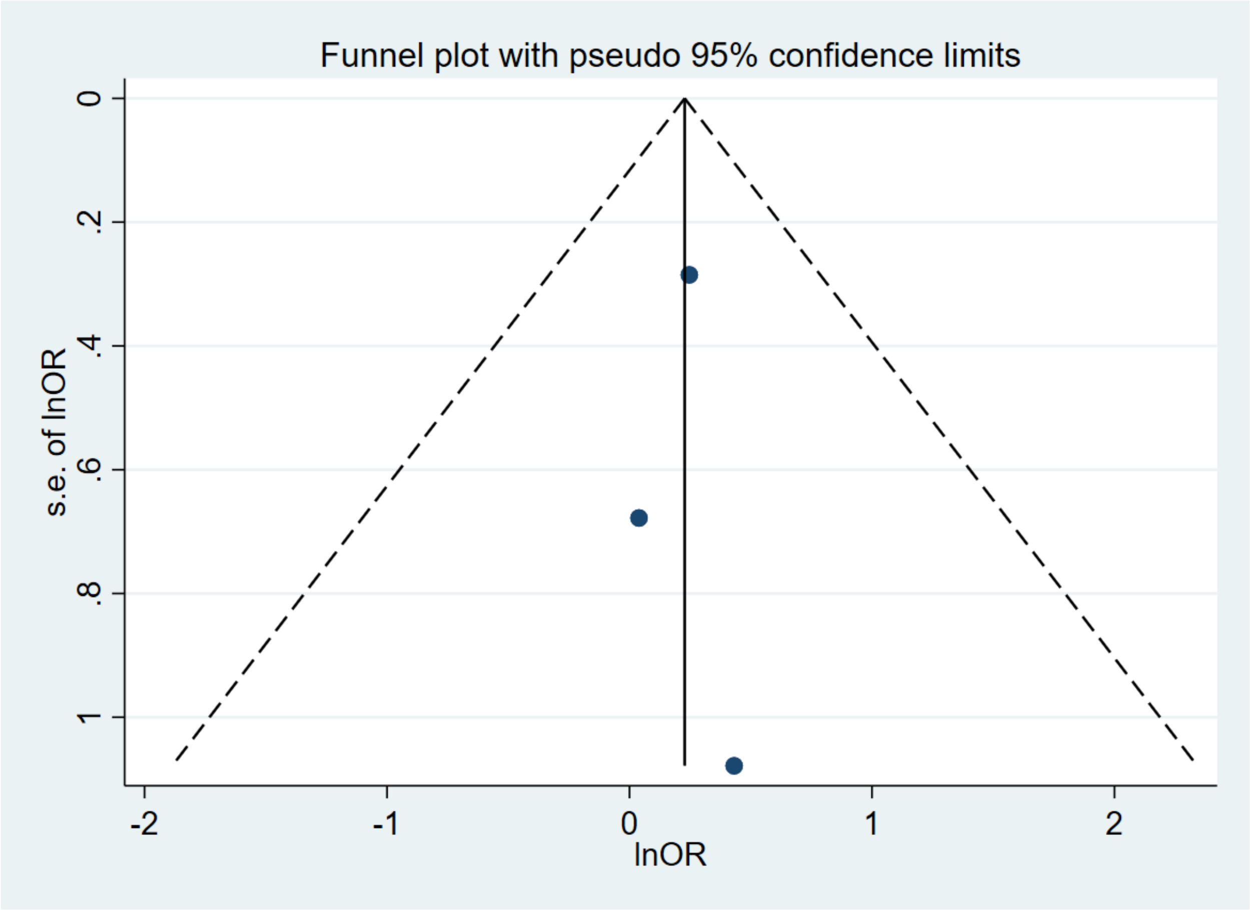

Supplementary figure 16

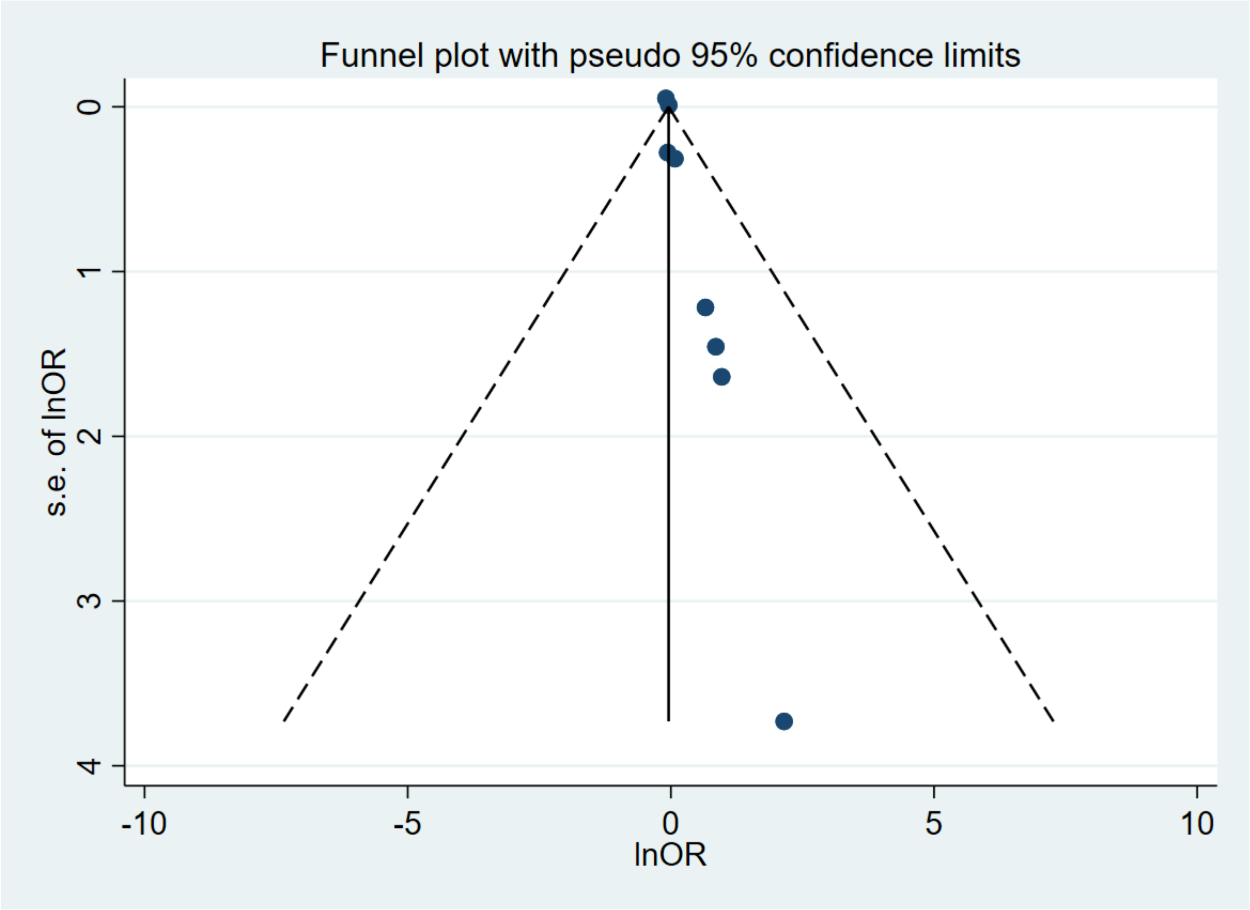

Supplementary figure 17

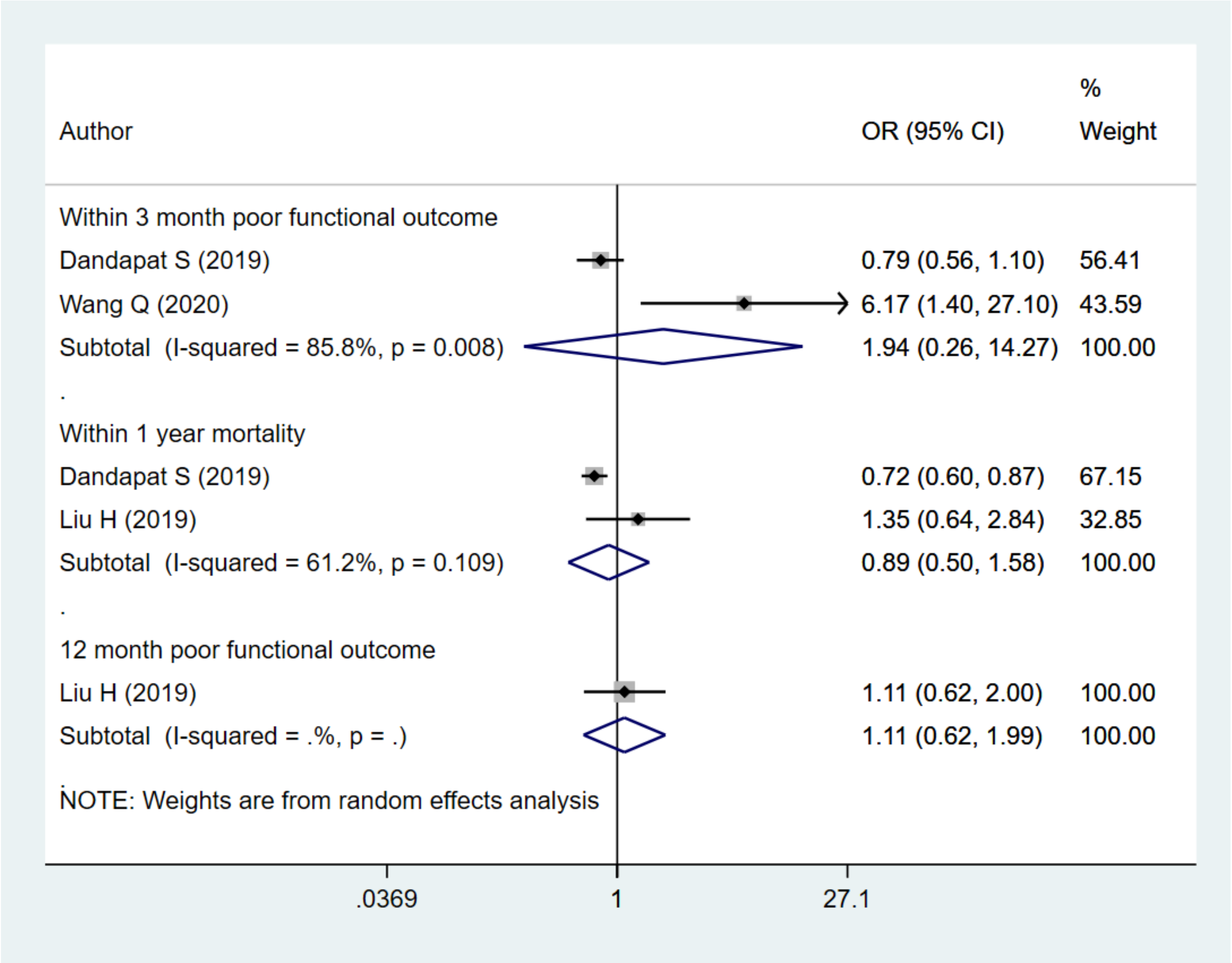

Supplementary figure 18

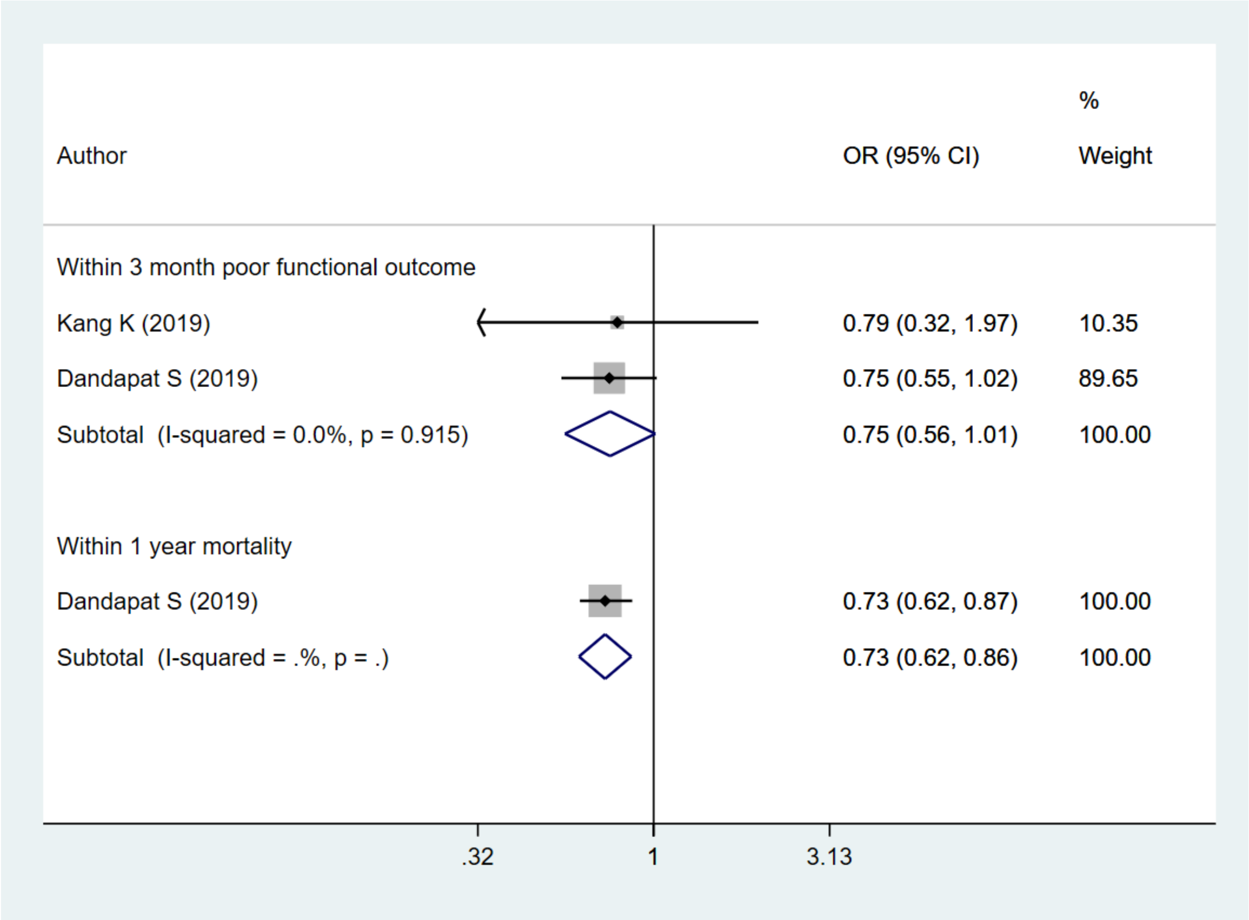

Supplementary figure 19

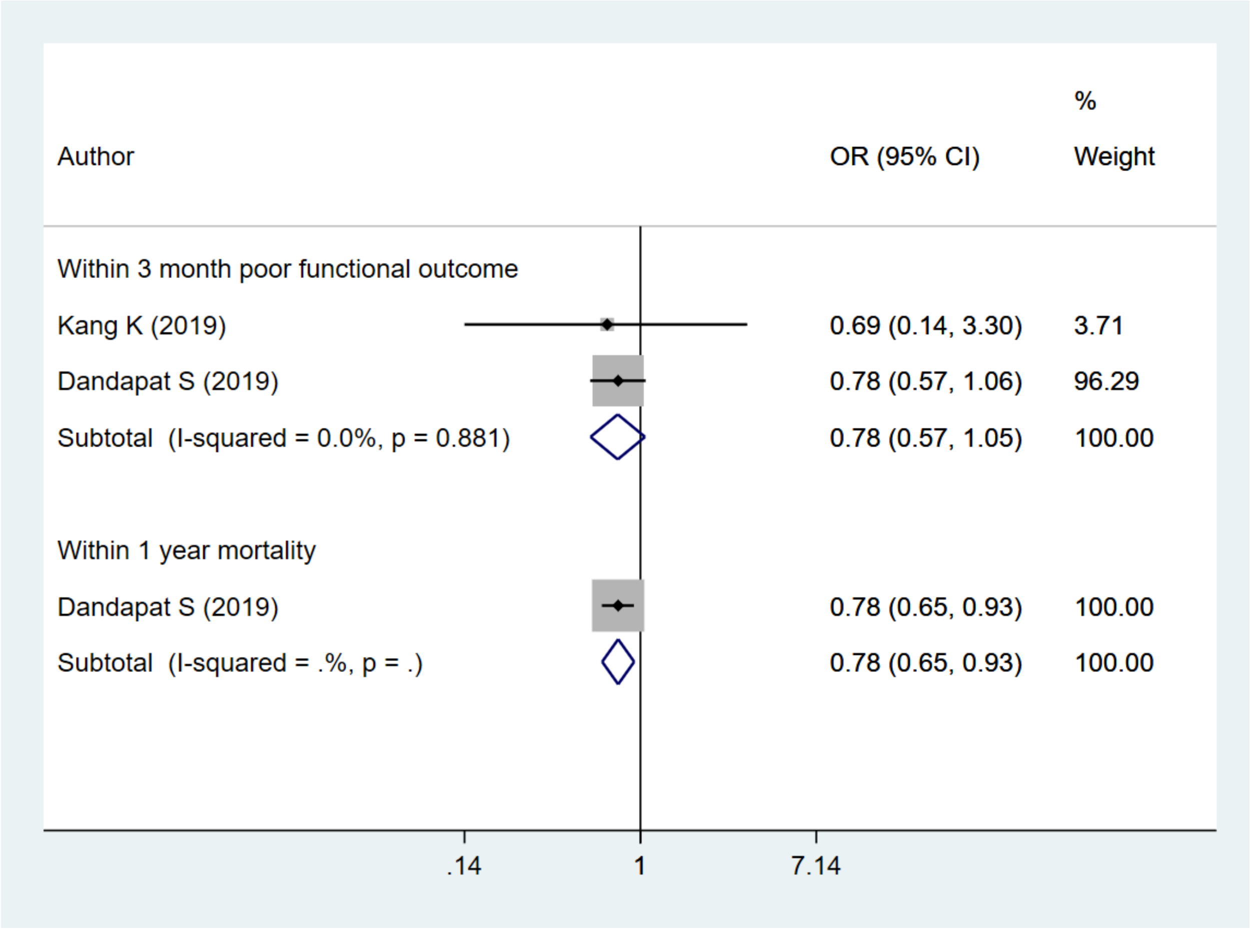

Supplement: Supplementary Figure 1 — Funnel plot for publication bias with respect to HbA1c (continuous) and mortality within 1 year of admission for acute ischemic stroke. [file Data_Sheet_1.PDF]
