# Supplementary material for: Glycated Hemoglobin as a Marker for Predicting Outcomes of Patients With Stroke (Ischemic and Hemorrhagic): A Systematic Review and Meta-Analysis
Source: Front Neurol. 2021 Mar 31;12:642899. doi: 10.3389/fneur.2021.642899 (PMC8044393; doi:10.3389/fneur.2021.642899)
Supplement: Supplementary Table 1 — Search strategy for identification of studies to be included in the review. [file Table_1.DOCX]

**Supplemental table 1. Search strategy for identification of studies to be included in the review**

| **Search strategy**  #1 (HbA1c OR glycated hemoglobin OR glycated haemoglobin OR glycemic control)  #2 (stroke OR acute ischemic stroke OR ischemic stroke OR hemorrhagic stroke OR haemorrhagic stroke)  #3 (mortality OR death OR functional outcome OR neurological complication OR adverse events OR prognosis)  #4 (#1 AND #2 AND #3)  #5 (Addresses[ptyp] OR Autobiography[ptyp] OR Bibliography[ptyp] OR Biography[ptyp] OR pubmed books[filter] OR Case Reports[ptyp] OR Congresses[ptyp] OR Consensus Development Conference[ptyp] OR Directory[ptyp] OR Duplicate Publication[ptyp] OR Editorial[ptyp] OR Systematic reviews OR Meta analysis OR Festschrift[ptyp] OR Guideline[ptyp] OR In Vitro[ptyp] OR Interview[ptyp] OR Lectures [ptyp] OR Legal Cases[ptyp] OR News[ptyp] OR Newspaper Article[ptyp] OR Personal Narratives [ptyp] OR Portraits[ptyp] OR Retracted Publication[ ptyp] OR Twin Study[ptyp] OR Video-Audio Media[ptyp])  #6 (#4 NOT #5) |
| --- |
