# Supplementary material for: Glycated Hemoglobin as a Marker for Predicting Outcomes of Patients With Stroke (Ischemic and Hemorrhagic): A Systematic Review and Meta-Analysis
Source: Front Neurol. 2021 Mar 31;12:642899. doi: 10.3389/fneur.2021.642899 (PMC8044393; doi:10.3389/fneur.2021.642899)
Supplement: Supplementary Table 2 — Author's judgements about study quality using the adapted Ottawa-Newcastle Risk of Bias Assessment tool. [file Table_2.DOCX]

**Supplementary table 2. Author’s judgements about study quality using the adapted Ottawa-Newcastle Risk of Bias Assessment tool**

|  | Diprose WK (2019) | Jing J (2016) | Wang H (2018) | Zhang G (2018) | Yang CJ (2017) | Wu S (2014) | Lattanzi S (2016) | Sunanda T (2016) | Choi KH (2018) | Hjalmarsson C (2014) | Kamouchi M (2011) | Rocco A (2013) |
| --- | --- | --- | --- | --- | --- | --- | --- | --- | --- | --- | --- | --- |
| Representativeness/appropriateness of participant selection  Random or consecutive recruitment=Y  Convenience sample=N  Not reported or unclear | Y | Y | Y | Y | Y | Y | Y | Y | Y | Y | Y | Y |
| Control for baseline differences in cohorts  Similarity of groups at baseline or adjustment in analyses=Y  No attempt to control or adjust=N  Not reported=NR | Y | Y | N | Y | Y | Y | Y | Y | N | Y | Y | Y |
| Loss to follow-up  Explanation provided for loss of participants and/or intention to treat=Y  No explanation =N | Y | Y | N | Y | Y | Y | Y | Y | Y | Y | Y | Y |
| Masking of exposure to outcomes assessor  Description of masking=Y  No masking or no description =N | Y | Y | Y | Y | Y | Y | Y | Y | Y | Y | Y | Y |
| Ascertainment of condition  Description of ascertainment/diagnostic criteria=Y  No description or patient self-report=N | Y | Y | Y | Y | Y | N | Y | Y | Y | N | Y | Y |
| Documentation of other treatment modalities  Documentation=Y  No documentation=N | Y | Y | Y | N | Y | Y | Y | N | Y | Y | Y | N |
| Extent to which valid outcomes are described  Adequate description of outcome=Y  Insufficient detail regarding outcome or follow-up time=N | Y | Y | Y | Y | Y | Y | Y | N | Y | Y | Y | Y |
| Prespecification of harms, mode of harms collection  Description of a list of harms assessed or monitoring=Y  No such description or passive harms collection=N  No adverse events reported=NA | Y | Y | Y | N | Y | N | Y | N | Y | N | Y | N |
| Financial Conflict of interest (COI)  Funding source reported=Y  Funding source not reported=N | N | Y | Y | N | Y | N | Y | N | Y | N | Y | N |

**Supplementary table 2 continued. Author’s judgements about study quality using the adapted Ottawa-Newcastle Risk of Bias Assessment tool**

|  | Masrur S (2015) | Gao Y (2016) | Lei C (2014) | Kang K (2019) | Dandapat S (2019) | Koga M (2015) | Zhang X (2018) | Zhang G (2015) | Liu H (2019) | Wang Q (2020) |
| --- | --- | --- | --- | --- | --- | --- | --- | --- | --- | --- |
| Representativeness/appropriateness of participant selection  Random or consecutive recruitment=Y  Convenience sample=N  Not reported or unclear | Y | Y | Y | Y | Y | Y | Y | Y | Y | Y |
| Control for baseline differences in cohorts  Similarity of groups at baseline or adjustment in analyses=Y  No attempt to control or adjust=N  Not reported=NR | Y | Y | Y | Y | Y | Y | Y | Y | Y | Y |
| Loss to follow-up  Explanation provided for loss of participants and/or intention to treat=Y  No explanation =N | Y | Y | Y | Y | N | Y | Y | Y | Y | Y |
| Masking of exposure to outcomes assessor  Description of masking=Y  No masking or no description =N | Y | Y | Y | N | Y | Y | Y | Y | Y | Y |
| Ascertainment of condition  Description of ascertainment/diagnostic criteria=Y  No description or patient self-report=N | Y | Y | Y | Y | Y | Y | Y | Y | Y | Y |
| Documentation of other treatment modalities  Documentation=Y  No documentation=N | Y | Y | Y | N | Y | Y | Y | N | Y | Y |
| Extent to which valid outcomes are described  Adequate description of outcome=Y  Insufficient detail regarding outcome or follow-up time=N | Y | Y | Y | Y | Y | N | Y | Y | Y | Y |
| Prespecification of harms, mode of harms collection  Description of a list of harms assessed or monitoring=Y  No such description or passive harms collection=N  No adverse events reported=NA | Y | Y | Y | N | Y | Y | N | N | Y | Y |
| Financial Conflict of interest (COI)  Funding source reported=Y  Funding source not reported=N | Y | N | Y | Y | Y | Y | N | Y | Y | N |
